# Exploring AAV‐Mediated Gene Therapy for Inner Ear Diseases: from Preclinical Success to Clinical Potential

**DOI:** 10.1002/advs.202408397

**Published:** 2025-06-20

**Authors:** Fan Wu, Wuhui He, Yun Xiao, Qiong Wu, Qing Zhang, Tiesong Zhang, Lei Xu, Xinyi Pang

**Affiliations:** ^1^ Department of Pathology and Laboratory Medicine The Medical University of South Carolina Walton Research Building Room 403‐E Charleston SC 29425 USA; ^2^ Department of Otolaryngology Sun Yat‐sen Memorial Hospital Sun Yat‐sen University Guangzhou Guangdong 510000 China; ^3^ Department of Otolaryngology–Head and Neck Surgery Shandong Provincial ENT Hospital Shandong University Jinan Shandong 250000 China; ^4^ Department of Otorhinolaryngology–Head and Neck Surgery Xinhua Hospital Shanghai Jiaotong University School of Medicine Shanghai, China/Ear Institute, Shanghai Jiaotong University School of Medicine, Shanghai China/Shanghai Key Laboratory of Translational Medicine on Ear and Nose Diseases Shanghai 200001 China; ^5^ Yunnan Key Laboratory of Children's Major Disease Research Yunnan Province Clinical Research Center for Children's Health and Disease Yunnan Institute of Pediatrics Kunming Key Laboratory of Children Infection and Immunity Kunming Children's Hospital Kunming Yunnan 650228 China; ^6^ Department of Otolaryngology–Head and Neck Surgery Shandong Provincial ENT Hospital Shandong University Jinan Shandong 250022 China; ^7^ College of Food Science and Engineering Nanjing University of Finance and Economics Nanjing 210023 China

**Keywords:** AAVs, acquired hearing loss, cochlear, gene therapies, genetic hearing loss, inner ears, sensory hair cells

## Abstract

Hearing loss imposes a significant global health burden and often results from genetic factors and various external influences, such as noise exposure and the use of ototoxic drugs. Recent advancements in adeno‐associated virus (AAV)‐mediated gene therapy offer promising new avenues for the treatment of inner ear diseases. Clinical trials of AAV‐mediated gene therapies show impressive preliminary results, although further research is needed to evaluate the safety and long‐term effects of these therapies. Preclinical AAV‐mediated gene therapy is notable for its high transduction efficiency and safety. In this article, the development of AAV‐mediated gene therapies is reviewed for inner ear diseases, categorizing these therapies by their strategies for treating hereditary hearing loss, including gene replacement and cluster regularly interspaced short palindromic repeat‐based methods. AAV‐mediated hair cell regeneration therapy is also reviewed for acquired hearing loss, as well as methods to prevent acquired hearing loss. In this article, it is hoped to provide a comprehensive overview of recent progress in AAV‐mediated gene therapy and its future potential, thereby highlighting the importance of this therapy for researchers and clinicians in the field.

## Graphical abstract

Current preclinical studies of AAV‐mediated gene therapy explore different strategies based on the characteristics of inner ear diseases. For genetic hearing loss, approaches include the replacement of a “good gene,” removal of a “bad gene,” or direct correction of mutations through base editing. For acquired hearing loss, strategies such as pre‐traumatic intervention and post‐traumatic hair cell regeneration are being developed.

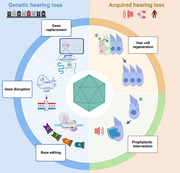

## 1 Introduction

One of the main functions of the cochlea is the generation of acoustic signals. The maintenance of normal cochlear function depends on the overall integrity of its structure, including its sensory hair cells, supporting cells (SCs), sensory neurons, and the structures necessary to maintain the endocochlear potential (EP). Approximately half of severe hearing loss (HL) cases are due to genetic factors, with more than 120 genes implicated in hereditary HL, thereby presenting a significant clinical challenge.^[^ ^1^ ^]^ Additionally, numerous internal and external damaging factors, such as noise exposure, ototoxic drug use, and chronic infections, can accumulate and contribute to cochlear malfunction.^[^ ^2^ ^]^ Sensory hair cell dysfunction is a common pathological feature of both hereditary and acquired HL.^[^ ^3^, ^4^, ^5^ ^]^ Unlike those in avian species, cochlear sensory hair cells in mature mammals do not regenerate spontaneously, making severe damage to human sensory hair cells irreversible.^[^ ^6^ ^]^

HL currently imposes a significant social health burden worldwide, and both the prevention and treatment of HL are high‐priority health concerns.^[^ ^7^ ^]^ Sixty years ago, no effective treatments were available for severe to profound HL. However, the cochlear implant (CI), which is regarded as a great advance in modern medicine, was the first effective treatment for deafness. The function of a CI is to bypass damaged structures and directly stimulate sensory neurons in the auditory nerve with reprogrammed electrical signals. Despite inspiring progress in CI technology, challenges remain, such as difficulty listening in noisy environments or listening to multiple speakers.^[^ ^8^ ^]^ Furthermore, electrode insertion‐associated damage and intracochlear fibrosis significantly limit the efficacy of CIs.^[^ ^9^, ^10^, ^11^ ^]^ Nonetheless, in addition to CIs, limited alternatives are available that can effectively affect the prognosis of patients with severe to profound HL.

Gene therapy medicinal products (GTMPs) are innovative treatments that aim to treat or prevent diseases by changing the genetic material within a patient's cells. The first attempt to modify DNA in humans was performed in 1980, and the first successful gene transfer in humans was performed in 1990. GTMPs are used to treat a variety of genetic disorders, cancers, and other diseases that have a genetic component. They can correct defective genes, introduce new genes, or silence harmful genes.^[^ ^12^, ^13^, ^14^ ^]^

GTMPs work by delivering genetic material into cells using vectors such as viruses (e.g., lentiviruses, adeno‐associated viruses) or non‐viral methods (e.g., plasmid DNA, mRNA, and small interfering RNA).^[^ ^15^, ^16^ ^]^ Viral vectors are highly efficient at delivering genetic material into cells. Certain viral vectors can be engineered to target specific cell types, enhancing the precision of gene delivery. However, some viral vectors can trigger immune responses, which may reduce their effectiveness and cause adverse effects. Additionally, producing viral vectors is relatively complex and costly.^[^ ^17^, ^18^, ^19^, ^20^ ^]^

In contrast, non‐viral methods generally pose a lower risk of immune responses and are often easier and cheaper to produce, but they typically have lower transfection efficiency. Moreover, non‐viral vectors often result in transient expression of the therapeutic gene, requiring repeated administrations. Non‐viral methods also generally lack the ability to target specific cell types as precisely as viral vectors.^[^ ^21^, ^22^, ^23^, ^24^ ^]^

In recent inner ear applications, gene therapy has been defined as the transfer of nucleic acids to target cells in the cochlea of an individual, resulting in a therapeutic effect.^[^ ^25^, ^26^, ^27^ ^]^ Compared to the relative insufficient transduction efficiency of non‐viral vectors, nonpathogenic virus‐mediated gene therapy has many advantages. Among these nonpathogenic viruses, adeno‐associated virus (AAV) has outstanding transduction efficiency and safety features.

AAV‐mediated gene therapy has become a hotspot in inner ear disease research and treatment, in both laboratory settings and clinical applications. Notably, impressive primary outcomes have been achieved in the most recent clinical trials of AAV‐mediated gene therapy for patients with autosomal‐recessive deafness 9 (DFNB9).^[^ ^28^, ^29^, ^30^ ^]^ Although many safety concerns and long‐term effects remain to be fully evaluated, AAV‐mediated gene therapy represents a significant advancement in the treatment of inner ear diseases. This therapy could be an alternative option to treat HL in the future.

In this article, we first introduce the basic features of AAVs and then review the development of AAV‐mediated inner ear gene therapy in recent years. Other vector‐mediated gene therapies for inner ear diseases will also be briefly introduced to provide a clear overview of this field. We categorize these studies mainly based on the strategy they adopt in treating hereditary HL, such as gene replacement and clustered regularly interspaced short palindromic repeat (CRISPR)‐based gene replacement, disruption, and correction. Additionally, we review the literature on AAV‐mediated hair cell regeneration and protection strategies for the treatment of acquired HL. Through this review, we hope to provide a clearer picture of the recent development of AAV‐mediated gene therapy for hearing disorders and to expand the vision of researchers and doctors regarding the future clinical applications of AAV‐mediated gene therapy for inner ear diseases.

## 2 Overview of AAVs

AAV, a member of the parvovirus family, was initially identified as a byproduct of adenovirus (AdV) by Atchison and colleagues in 1965^[^ ^31^ ^]^ and was further characterized by Hoggan et al. in 1966.^[^ ^32^ ^]^ As one of the smallest viruses, AAV possesses a capsid composed of 60 subunits of capsid proteins (VP1, VP2, and VP3), which encapsulate single‐stranded DNA (ssDNA) of 4.7 kilobases in size.^[^ ^33^ ^]^ The AAV genome encodes for 3 important genes, *Rep*, *Cap*, and *Aap*, which are necessary for replication and encapsidation.^[^ ^34^ ^]^ Variations in the capsid protein encoded by *Cap* result in diverse AAV serotypes, including the primary serotypes AAV1‐13 and their variants.^[^ ^35^, ^36^ ^]^

Another crucial feature of AAVs is their inverted terminal repeats (ITRs), which flank open reading frames and serve as packaging signals for ssDNA. Owing to the inability of AAVs to replicate independently, coinfection with a helper virus such as AdV is required for the efficient replication of AAVs in cells under natural conditions. Plasmids containing helper genes have been substituted for coinfection to mitigate the potential risk of helper virus infection.^[^ ^37^ ^]^

Recombinant AAV vectors are derived from natural AAVs. For AAV vectors, the ITR‐flanked genes of AAVs are replaced with an expression cassette. When coexisting with helper plasmids that provide structural (*Cap*) and nonstructural (*Rep*) viral proteins and virus helper genes, AAV vectors are replicated and packaged in producer cells, such as the 293T cell line, facilitating large‐scale production for applications in large animal models or human trials.^[^ ^38^ ^]^ AAVs commonly enter target cells through endocytosis, which is facilitated by the binding of AAVs to cell surface receptors or uptake via the Clathrin‐independent endocytosis pathway (CLIC/GEEC).^[^ ^37^, ^39^ ^]^ Another universal receptor shared among different serotypes was recently reported and is known as KIAA0319L or AAVR, the knockout of which largely blocks AAV infection in vivo and in vitro.^[^ ^40^ ^]^ Once inside target cells, the AAV genome can persist episomally in the nucleus and support stable, long‐term transcription, making it promising for gene delivery and transgenic therapy.

Before AAV was widely applied, other viral vectors, such as adenovirus and lentivirus, were considered promising for gene therapy. Adenovirus (Adv) has greater packaging capacity (≈8.5 kb) compared to AAV (≈4.5 kb).^[^ ^41^ ^]^ AdV can infect both inner hair cells (IHCs) and outer hair cells (OHCs) from the organ of Corti, SCs, and hair cells from the utricle in neonatal mice.^[^ ^42^ ^]^ AdV‐based gene therapies have been shown to treat inner ear diseases such as transient cochlear ischemia damage, D‐gal‐induced damage, and kanamycin‐induced ototoxicity.^[^ ^43^, ^44^, ^45^ ^]^ However, considering the pathogenic features of AdV, which can induce toxicity and immune responses, AdV‐based gene therapies have not been developed as quickly as AAV‐based gene therapies have in recent years. Lentivirus (Lv) also has a relatively large packaging capacity (≈10 kb) and is another suitable vector for carrying large genes.^[^ ^46^ ^]^ The third generation of Lvs has been shown to have better safety and efficiency, along with a broad host range.^[^ ^47^ ^]^ However, genes delivered by lentiviruses are integrated into the host genome, and this reverse transcription feature of Lvs poses a risk of insertional mutagenesis.^[^ ^48^, ^49^ ^]^ In addition, Lvs have been reported to cause immunogenicity and ototoxicity in inner ear applications.^[^ ^50^ ^]^ Due to their size and the presence of viral proteins on their surface, lentiviruses can trigger a strong immune response, including innate and adaptive immune systems. This activation leads to the production of antibodies and cytotoxic T cells, which in turn results in the clearance of the vector and infected cells.^[^ ^51^, ^52^, ^53^ ^]^ In terms of safety, AAV has more advantages than these two viral vectors.

AAV‐based transgenic therapy was first applied in 1996 in a clinical trial for the treatment of cystic fibrosis.^[^ ^54^ ^]^ Significant progress has also been achieved in treating hemophilia by administering AAV‐based gene therapy to affected patients.^[^ ^55^, ^56^ ^]^ To date, several AAV‐based drugs have been approved for clinical use.^[^ ^57^ ^]^ Hundreds of AAV‐based transgenic therapies target genes in various organs, such as the brain, eyes, liver, and muscles.^[^ ^58^, ^59^, ^60^ ^]^ Although the application of AAV‐based transgene therapy for inner ear diseases has slightly lagged, this therapy has developed rapidly in the past five years. As the most promising platform for transgenic therapy, especially for monogenic diseases, AAV still has both merits and flaws. Below, we summarize the features of AAVs that make them useful for gene therapy, as well as the development of AAV‐mediated gene therapies in recent years.

## 3 Advantages and Recent Developments of AAVs

Compared with transduction with other viral vectors, AAV‐mediated transduction has numerous advantages, including high infection efficiency, stable and long‐term gene expression, relatively low immunogenicity, and increased safety.^[^ ^37^, ^61^, ^62^, ^63^, ^64^, ^65^ ^]^ Notably, the diverse array of AAV serotypes and their variants, characterized by high tropism specificity, allows the targeting of a broad range of host cells and organs, facilitating the development of tailored serotypes for specific therapeutic applications.^[^ ^66^, ^67^, ^68^ ^]^

AAVs have been reported to be nonpathogenic in target cells and organs.^[^ ^69^, ^70^ ^]^ Naturally, AAVs are widely distributed among various hosts, including mammals and humans.^[^ ^68^, ^71^ ^]^ Upon infection, AAVs, as defective viruses, persist in a latent state as episomes without replication capacity unless coinfection with helper viruses is performed.^[^ ^72^, ^73^ ^]^ Furthermore, the asynchronous replication between the AAV genome and the host genome leads to the dilution of AAV in actively dividing cells.^[^ ^37^, ^74^ ^]^ Additionally, the majority of the AAV genome is replaced with the gene of interest to create recombinant AAV, resulting in a diminished replication capacity due to the removal of the *Rep* and *Cap* genes.^[^ ^75^, ^76^, ^77^ ^]^ Therefore, the use of AAV vectors is considered a safe approach for gene transduction (classified as Risk Grade 1 by the National Institutes of Health).

The immune response of host subjects can be elicited by the viral capsid, viral genome, or proteins translated from the transgene.^[^ ^78^, ^79^ ^]^ Compared with lentiviruses (Lvs) and AdVs, AAVs exhibit less immunogenicity, primarily because of the small size of their capsids, thereby contributing to their safety for host subjects.^[^ ^63^, ^64^ ^]^ The innate immune responses triggered by capsid proteins are related to the dose and route of AAV administration, as well as the age or condition of the subject.^[^ ^80^, ^81^ ^]^ The low immunogenicity of AAVs minimizes the adverse symptoms associated with host immune responses, such as fever or hypersensitivity reactions, following AAV administration.^[^ ^82^, ^83^ ^]^ Furthermore, the low immunogenicity of AAVs hinders their detection and elimination by the host immune system, resulting in higher concentrations and longer durations of transgene expression in the target organs.^[^ ^63^, ^84^, ^85^ ^]^

The episomal form of the AAV genome within the nucleus of host cells also facilitates long‐term AAV persistence and stable expression of the transgene, especially in stably infected cells.^[^ ^86^, ^87^ ^]^ The duration of expression ranges from several months to ten years, depending on the target organ or cell, delivery method, and transgene sequence.^[^ ^55^, ^88^, ^89^ ^]^ In inner ear experiments, numerous animal studies have shown that AAV‐mediated expression can persist for more than 10 months in mice.^[^ ^90^, ^91^, ^92^ ^]^ Since most mouse stains have a lifespan longer than 2 years, longer observations of AAV transgene expression are still warranted.^[^ ^93^ ^]^ Long‐term stable expression of the transgene benefits sustained treatment outcomes. However, due to its low integration rate, the efficiency of transgene expression may decrease at later stages.^[^ ^94^, ^95^, ^96^ ^]^ Clinical trials have reported a decrease in the effectiveness of AAV treatment in patients with hemophilia and retinal diseases.^[^ ^97^, ^98^, ^99^, ^100^, ^101^ ^]^ Based on these outcomes, long‐term observations are needed for AAV‐mediated inner ear gene therapy.

AAVs exhibit different tissue tropisms largely depending on their serotypes. This feature may be due to the unique interactions between AAV capsid proteins and receptors on the host cell surface.^[^ ^39^, ^102^ ^]^ Currently, there are more than 23 glycan receptors identified that serve different serotypes of AAV, such as sialic acid and heparan sulfate proteoglycan (HSPG).^[^ ^103^, ^104^, ^105^, ^106^ ^]^ The distribution of these receptors in different organs may largely determine the tropism features of different AAV serotypes.^[^ ^66^ ^]^ Additionally, in 2016, Pillay et al. identified KIAA0319L (known as AAVR) as a universal receptor for AAV infection.^[^ ^40^ ^]^ Based on this finding, in 2023, Zengel et al. developed a mouse model to conditionally express AAVR in different organs, successfully achieving controllable AAV tropism.^[^ ^107^ ^]^ Our recent findings suggest that AAVR is critical for AAV infection in sensory hair cells. Decreased AAVR expression in adult mice OHCs contributes to the low transduction efficiency of AAV2, Anc80L65, and AAV2.7m8. Conditional overexpression of AAVR can effectively restore AAV infection sensitivity in adult mice OHCs.^[^ ^108^ ^]^

The feature of organ tropism increases the transduction efficiency in target cells while minimizing nontherapeutic effects on other organs, leading to more effective and safer treatment outcomes.^[^ ^109^, ^110^ ^]^ To date, the development of AAV vectors has increased their tissue tropism within the inner ear, resulting in greater transduction efficiency.^[^ ^111^, ^112^ ^]^ However, other factors, such as the routes of administration, influence therapeutic outcomes and safety. For example, the systemic administration of AAV9 has been reported to cause liver toxicity.^[^ ^113^, ^114^ ^]^ Therefore, a local delivery method is preferred to avoid systemic off‐target side effects. Below, we summarize the development of AAVs in the inner ear in recent years.

### 3.1 AAV Capsid Development

Currently, at least 13 primary serotypes and hundreds of variants have been identified.^[^ ^115^ ^]^ Among these, AAV1, AAV2, and AAV9 are the most utilized primary types for inner ear transduction, achieving higher transduction efficiency in sensory hair cells than other primary serotypes.^[^ ^116^, ^117^, ^118^ ^]^ Additionally, the use of genetically engineered capsids through the insertion of specific peptides, point mutations, and ancestral reconstruction based on the original capsid has widened the spectrum and transduction efficiency of target cells.^[^ ^39^, ^119^ ^]^ The aim of capsid engineering is to modify capsids using various strategies to increase transduction outcomes.

In recent years, several key strategies have been developed for AAV capsid engineering. For example, directed evolution involves creating large libraries of capsid variants through random mutations and then selecting those with desired properties, such as improved tissue targeting or immune evasion.^[^ ^120^, ^121^ ^]^ Another strategy, peptide insertion, involves inserting peptides into specific regions of the capsid to create variants with new targeting capabilities. This method has been used to increase the transduction efficiency in specific tissues, such as those of the central nervous system.^[^ ^122^, ^123^, ^124^, ^125^, ^126^ ^]^ Finally, rational design uses structural and functional knowledge of AAV capsids to generate specific, targeted modifications. For example, researchers might alter surface‐exposed loops to improve receptor binding or evade neutralizing antibodies.^[^ ^127^, ^128^, ^129^ ^]^ These methods can accelerate the discovery of effective capsid modifications.

The cochlea, a key organ in the auditory system, contains many neurons and receptor cells that play crucial roles in hearing. Many AAV serotypes exhibit similar organ tropism between the cochlea and the CNS.^[^ ^130^, ^131^ ^]^ Sensory hair cells and spiral ganglion neurons are the two main functional cell types in the inner ear responsible for sound signal generation and transmission.^[^ ^132^ ^]^ Additionally, the stria vascularis epithelium is important for maintaining the EP.^[^ ^133^ ^]^ Among these cell groups, mutations or direct trauma to sensory hair cells are the most common causes of sensory hearing loss. Currently, most AAV‐mediated gene therapies for hereditary and acquired hearing loss target sensory hair cells. Conversely, studies focusing on regenerating new sensory hair cells are more likely to target SCs with differential potential.^[^ ^30^, ^112^ ^]^ Below, we introduce some of the representative AAV variants that have been developed in recent years that stand out, especially in pre‐clinical inner ear gene therapy.

PHP.B Benjamin and colleagues employed a capsid selection method in 2016, to overcome the BBB and increase the transduction efficiency of AAV in the CNS, resulting in the generation of a novel serotype of AAV named AAV‐PHP.B, derived from the capsid of AAV9. AAV‐PHP.B exhibits a significantly greater transduction efficiency in all regions of the CNS, at least 40 times greater than that of AAV9.^[^ ^134^ ^]^ Bence György et al. subsequently evaluated the transduction efficiency of AAV‐PHP.B in the mouse cochlea. They reported that the transduction efficiency was 60%–80% for IHCs and 30–40% for OHCs in neonatal mice.^[^ ^135^ ^]^ In adult mice, IHCs can be easily infected with PHP.B (apex: 90%; middle: 100%; and base: 100%), OHCs are significantly resistant to AAV‐PHP.B transduction, with no detectable GFP signal in OHCs. Moreover, the transduction efficiency of AAV‐PHP.B in Tuj‐1‐positive spiral ganglion neurons (SGNs) was also high in adult mice.^[^ ^136^, ^137^ ^]^ To date, transgenic therapy mediated by AAV‐PHP.B has been successfully applied to restore hearing in various mouse models.^[^ ^90^, ^138^, ^139^, ^140^, ^141^, ^142^ ^]^ Furthermore, AAV9‐PHP.B has been extensively validated to transduce cochlear hair cells and vestibular hair cells in fetal explants, as well as vestibular hair cells in adult explants derived from human inner ear sensory epithelia.^[^ ^143^ ^]^ While PHP.B is highly effective in some mouse models such as C57BL/6 and FVB/NJ, and has proven ineffective in nonhuman primates (NHPs) because of the species‐specific receptor LY6a expressed on brain endothelial cells.^[^ ^144^, ^145^ ^]^ Mouse strains such as BALB/cJ, which lack LY6a expression, also present a low PHP.B transduction efficiency.^[^ ^146^ ^]^ The expression of LY6a enables PHP.B to be transported across the blood–brain barrier (BBB). Therefore, the expression of LY6a must be tested in the human inner ear when considering PHP.B as a serotype for human translation. In addition to its effect on sensory hair cells, PHP.B reportedly has a 50% higher transduction rate in Sox2‐positive SCs.^[^ ^147^ ^]^

AAV2.7m8 was first reported in 2013, and AAV2.7m8 emerged as a variant of AAV2 aimed at overcoming biological barriers to increase gene delivery to the outer retina.^[^ ^148^ ^]^ Kevin Isgrig et al. subsequently demonstrated that AA2.7m8 has high transduction efficiency in the cochleae of neonatal mice, reaching 84.1% for IHCs and 83.1% for OHCs.^[^ ^149^ ^]^ Notably, AA2.7m8 also achieved impressive transduction efficiency in the sensory hair cells of adult mice—84.5% for IHCs and 74.9% for OHCs. Additionally, AAV2.7m8 infects SCs with high efficiency (inner pillar cells: 86.1% and inner phalangeal cells: 61.4%) but vestibular hair cells with less efficiency (≈30%).^[^ ^149^, ^150^ ^]^ In addition to inner ear diseases, transgene delivery by AAV2.7m8 has been shown to rescue photoreceptor‐related disease phenotypes in animal models of Leber congenital amaurosis (LCA) and retinoschisis and to treat choroidal neovascularization (CNV) in a laser‐induced model of CNV.^[^ ^151^, ^152^ ^]^ Several clinical trials have adopted the AAV2.7m8 serotype for the treatment of retinal diseases in humans (NCT03326336, PIONEER; NCT04945772, RESTORE).^[^ ^17^, ^148^ ^]^ These promising results support the use of AAV2.7m8 as an ideal serotype with high transduction efficiency and safety.

Anc80 The AAV‐Anc80 serotype, initially reported by the Grousbeck Gene Therapy Centre in 2015, was developed via ancestral sequence reconstruction techniques to trace the genetic lineage of AAV serotypes 1, 2, 8, and 9. Following rigorous screening, the 65th Anc80Lib clone of AAV‐Anc80 emerged as a promising candidate, exhibiting robust gene transduction efficiency in the liver, muscle, and retina.^[^ ^153^ ^]^ Lukas et al. subsequently observed a relatively low dose of AAV‐Anc80L65, a derivative of AAV‐Anc80, achieves complete transduction in both IHCs and OHCs (≈100%) in neonatal mice, outperforming AAVs 1, 2, 6, and 8.^[^ ^154^ ^]^ The high infection efficiency of AAV‐Anc80 has been validated in numerous subsequent studies.^[^ ^116^, ^117^, ^155^, ^156^, ^157^, ^158^ ^]^ In adult mice, Yu Zhao et al. reported a marked difference in the transduction efficiency of AAV‐Anc80 in OHCs, with a 52.81% transduction efficiency in the apical region but 0% in the base region, but IHCs remain highly sensitive to Anc80 transduction (≈100%).^[^ ^136^ ^]^ The variation in efficiency in different regions of the basilar membrane may be due to the injection location, affecting the concentration of AAV in the perilymph. Additionally, AAV‐Anc80L65 has displayed favorable transduction efficiency in spiral ganglion neurons (≈95%), vestibular ganglion neurons (≈70%), and vestibular hair cells (≈60%), when injected into neonates.^[^ ^159^ ^]^ However, low to moderate transduction efficiency (≈50%) of AAV‐Anc80 for SCs in the cochlea has also been reported.^[^ ^160^ ^]^

AAV8BP2 Therese Cronin et al. introduced the AAV8BP2 serotype in 2014 to achieve robust optogenetic transgene expression in retinal bipolar cells, employing a mutation‐based approach derived from the AAV8 capsid.^[^ ^161^ ^]^ In 2019, Kevin Isgrig et al. administered AAV8BP2‐GFP into the inner ears of mice via the posterior semicircular canal approach, and they reported transduction efficiencies of 55.7% for IHCs, 44.1% for OHCs, and 34.2% for vestibular hair cells, with negligible transduction in SCs in neonatal mice.^[^ ^149^ ^]^ Additionally, AAV8BP2 has been reported to effectively transduce marginal cells (40.9%), intermediate cells (26%) in the stria vascularis, and epithelial cells of the endolymphatic sac (19.8%) in neonatal mice.^[^ ^155^ ^]^ This unique feature of AAV8BP2 was exploited in a subsequent study to deliver genes to cochlear lateral wall cells.^[^ ^162^ ^]^ A combination of AAV‐8BP2 and AAV‐2.7m8 has been utilized to transduce diverse cell populations within the cochlea.^[^ ^162^ ^]^ However, potential adverse effects on auditory and vestibular functions were reported following an AAV8BP2 injection into the inner ear; these adverse effects may be attributable to the immunogenicity of AAV8BP2.^[^ ^149^ ^]^

AAV‐DJ Utilizing an adapted DNA family shuffling technology derived from the five initial serotypes of AAV 2, 4, 5, 8, and 9, Dirk Grimm et al. generated AAV‐DJ in 2008 with the goal of increasing AAV transduction efficiency in the liver.^[^ ^163^ ^]^ Two variants, AAV‐DJ8 and AAV‐DJ9, were subsequently developed through modifications targeting specific residues of AAV8 and AAV9, respectively.^[^ ^164^ ^]^ Investigations into the transduction efficiency of AAV‐DJ in the inner ears of neonatal mice revealed ≈a 50% transduction efficiency for IHCs and a 90% transduction efficiency for OHCs, except in the base region, where it was 37%. Notably, AAV‐DJ8 achieved infection rates of 59%, 24%, and 10% in IHCs located in the apical, middle, and basal cochlear turns, respectively, with minimal transduction observed in OHCs.^[^ ^165^ ^]^ Furthermore, transduction in the vestibules was observed in neonatal mice following AAV‐DJ or AAV‐DJ8 injection (the specific efficiency rate was not provided).^[^ ^165^ ^]^ Another study documented a 74% transduction efficiency for SCs in cochlear explants in vitro when AAV‐DJ was used.^[^ ^160^ ^]^

AAV‐KP1 Efficient transduction (comparable to that of the AAV‐DJ capsid) has been observed across various murine and human cell lines for the AAV‐KP1 capsid, which was identified from screening of a shuffled AAV capsid library.^[^ ^163^, ^166^ ^]^ A subsequent inner ear study conducted by Ksenia et al. revealed that AAV‐KP1 can achieve almost 100% transduction efficiency in most SCs (Deiters` cells, outer pillar cells, inner pillar cells, and inner phalangeal cells) within five days after injection and achieves optimal transduction efficiencies in IHCs (92.9%) and OHCs (88.2%) 20 days after injection into neonates. Additionally, SGN transduction by AAV‐KP1 was detected, with an efficiency ranging from 34% to 68.7% (from the base to the apex).^[^ ^167^ ^]^

AAV‐ie In 2019, a variant serotype, AAV‐ie (AAV‐inner ear), was developed to increase the transduction efficiency of AAV in inner ear SCs. This development of this variant involved the insertion of a cell‐penetrating peptide (CPP)‐like peptide (DGTLAVPFK) from the AAV PHP.eB vector into the VP1 capsid of AAV‐DJ.^[^ ^160^ ^]^ High transduction efficiency of AAV‐ie in cochlear SCs was observed both in vitro (≈90%) and in vivo (≈80%).^[^ ^168^, ^169^ ^]^ Importantly, AAV‐ie‐transduced human utricle samples showed high transduction efficiency in utricular SCs and hair cells, and ≈93% of utricular SCs and 76% of hair cells could be transduced by AAV‐ie.^[^ ^160^ ^]^ In 2022, AAV‐ie‐K558R, a mutant type of AAV‐ie, was screened and identified, displaying superior efficiency to wild‐type AAV‐ie in cochlear SCs in the basal region (≈80%).^[^ ^169^ ^]^ Owing to its high transduction efficiency for SCs, AAV‐ie has primarily been adopted in regeneration studies that differentiate hair cells from SCs.^[^ ^170^, ^171^, ^172^, ^173^ ^]^ Additionally, AAV‐ie and AAV‐ie‐K558R exhibited high transduction efficiency in sensory hair cells. AAV‐ie has been reported to infect ≈100% of IHCs and ≈80% of OHCs in neonatal mice. AAV‐ie‐K558R has been reported to infect almost all IHCs and OHCs (≈100%).^[^ ^169^, ^174^, ^175^ ^]^

AAV‐S In 2021, AAV‐S was generated from AAV9 through random capsid library selection. AAV‐S can transduce various cochlear cell types in both mice and nonhuman primates.^[^ ^176^ ^]^ After injection into neonatal mice, AAV‐S transduced almost all IHCs (≈100%) and over half of the OHCs (apical: ≈75% and basal: ≈50%). In the inner ears of cynomolgus monkeys, AAV‐S efficiently transduced diverse cell populations, including Deiters' cells, phalangeal cells, pillar cells, Hensen's cells, Claudius cells, and sulcus cells (specific efficiency rates were not provided). In addition, AAV‐S was reported to achieve almost 100% infection in IHCs and a regional decrease of infection rate in OHCs (apical region: 100%; mid‐base region: 70%; and basal region: 30%).^[^ ^176^ ^]^

Exo‐AAVs Unlike the modulation of AAV capsids to increase receptor–capsid interactions, a new strategy involving exosome encapsulation has been developed. AAV‐associated exosomes (Exo‐AAVs) are AAV capsids encapsulated within exosomes secreted by the cells used to produce AAV. These Exo‐AAVs can be purified from the media of vector‐producing cell lines.^[^ ^177^ ^]^ In the context of exosomes, Exo‐AAVs have a superior transduction efficiency and the potential to regulate specificity by modifying the ligands of the exosomes. Compared with conventional AAVs, Exo‐AAVs are more capable of crossing the BBB and penetrating the retina.^[^ ^178^, ^179^, ^180^ ^]^ One of the major clinical barriers for AAV application is the presence of neutralizing antibodies (NAbs). Although knowledge about NAbs is limited, they can bind directly to AAV and reduce its transduction efficiency.^[^ ^181^, ^182^ ^]^ Due to their lipid‐based structure, Exo‐AAVs are reported to be more resistant to NAbs than regular AAVs. This resistance increases the transduction efficiency and results in longer‐lasting effects, even in subjects undergoing retreatment.^[^ ^180^, ^183^, ^184^, ^185^ ^]^ In 2017, Bence György et al. reported that Exo‐AAV1‐GFP transduced up to 65% of IHCs and 50% of OHCs, whereas the conventional AAV1‐GFP vector transduced only ≈20% of both IHCs and OHCs. Additionally, they found that ≈95% of IHCs and OHCs were transduced with Exo‐AAV9, whereas 80% of IHCs and 60% of OHCs were transduced with regular AAV9.^[^ ^186^ ^]^

Magnetic AAVs The magnetic targeting of gene therapy products represents a novel and rapidly advancing field. In this technique, magnetizable nanoparticles are used to deliver and localize therapeutic agents precisely to the diseased area, ensuring safe and effective treatment.^[^ ^187^, ^188^, ^189^ ^]^ Subhendu Mukherjee developed a strategy in 2022 that involves attaching AAVs to magnetic molecules called SPIONs (superparamagnetic iron oxide nanoparticles) to increase the local tropism of AAVs in the inner ear while avoiding disruption of inner ear homeostasis. After the AAV‐SPION mixture was applied locally to the round window, a magnet was placed on the contralateral ear for 30 min. This method successfully achieves high sensory hair cell transduction efficiency in adult rats without disturbing hearing function (the specific efficiency rate was not provided).^[^ ^190^ ^]^

### 3.2 Development of AAV Promoters

In recent years, a variety of promoters have been studied for different purposes in inner ear therapy. Several ubiquitous promoters have been investigated to increase the overall infection rate in the inner ear without considering cell‐type specificity.^[^ ^191^, ^192^ ^]^ The cytomegalovirus (CMV) promoter, derived from cytomegalovirus, is the initial and most widely employed promoter in AAV‐based gene delivery systems for the inner ear. The CMV promoter has been shown to exhibit limited selective activity in specific cell populations within the cochlea.^[^ ^192^ ^]^

Another commonly used promoter in inner ear gene therapy is the CAG promoter, which is a fusion of the CMV enhancer and the chicken β‐actin (CBA) or rabbit β‐globin promoter. Compared with the CMV promoter, the CAG promoter has been reported to be more effective at driving transgene expression in SCs while also displaying promoter activity in IHCs and other cochlear cells.^[^ ^117^, ^138^, ^191^ ^]^ However, both the CMV and CAG promoters are constitutively expressed in organs other than the inner ear, potentially leading to nontarget transgene expression and adverse effects.^[^ ^193^, ^194^ ^]^ Similar issues are also encountered with other constitutive promoters, such as the CBA, elongation factor 1a (EF1a), and simian virus 40 (SV40) promoters. Moreover, the larger size of the CAG promoter restricts its application in transgenic therapies for the inner ear.^[^ ^195^ ^]^

Ubiquitous promoters, which lack cell type specificity, provide sustained, high‐level transgene expression across various cell types. However, they can result in toxic effects on nontargeted cells. Researchers have used specific promoters to drive transgene expression in targeted subpopulations of cochlear cells to mitigate this risk, suggesting their significant potential for advancing inner ear gene therapy.^[^ ^138^, ^140^, ^191^, ^196^, ^197^ ^]^ Among these promoters, the Myo15 promoter is notable because it selectively drives transgene expression in hair cells of the inner ear (patent no. US 2021/0388045 A1). The Myo15 promoter can efficiently drive GFP expression in both IHCs and OHCs of neonatal and adult mice, with limited expression in other inner ear organs, excluding vestibular hair cells.^[^ ^138^ ^]^ Notably, the Myo15 promoter has produced promising results in recent clinical trials of AAV‐based inner ear gene therapy.^[^ ^30^, ^198^ ^]^

Finally, efforts to design shorter promoter sequences while maintaining the functional characteristics of these promoters are also noteworthy. This approach enables the packaging of larger transgene sequences into AAVs. For example, Shaowei Hu et al.^[^ ^140^ ^]^ successfully truncated the Myo15 promoter from 1611 to 956 bp using a strategy called multiple vectors in one AAV. The truncated promoter retained its normal and hair cell‐specific activity, leaving more space for other elements to be packaged into the AAV.

### 3.3 Development of Surgical Approaches

The inner ear is a relatively enclosed space consisting of two functionally distinct organs: the vestibular system and the cochlea.^[^ ^199^ ^]^ The cochlea is divided into three unique fluid‐filled cavities. The scala vestibuli and scala tympani contain perilymph fluid, which is similar to cerebrospinal fluid. The scala media contains endolymph, which has a low concentration of Na+ ions and a high concentration of K+ ions.^[^ ^200^, ^201^, ^202^, ^203^ ^]^ The round and oval windows are located between the middle and inner ear. The RWM is located at the end of the scala tympani. In neonatal mice, the inner ear bones are soft and can be easily accessed with a microglass tube, causing minimal structural damage.^[^ ^204^, ^205^ ^]^ However, the bone ossifies with age, complicating AAV delivery into the inner ear.^[^ ^206^ ^]^ The selection of an appropriate approach can avoid injection‐associated damage while achieving sufficient infection efficiency in target cells. Below, we summarize several main AAV injection approaches that have been developed recently.

The round window membrane (RWM) approach has been reported as an ideal method for AAV delivery in neonatal mice.^[^ ^165^, ^207^, ^208^ ^]^ On the one hand, the round window is soft and easily recovers after injection in neonatal mice, making it less likely to cause injection‐associated inner ear trauma.^[^ ^209^ ^]^ On the other hand, the perilymphatic space is much larger than the endolymphatic space, making this approach more suitable for larger injection volumes.^[^ ^210^ ^]^ Researchers have reported that 60–80% transduction of IHCs and 40–70% transduction of OHCs can be achieved through the trans‐RWM injection of AAV9‐PHP.B in neonatal mice.^[^ ^135^ ^]^ Better results were obtained with the Anc80L65 serotype, which achieved ≈100% transduction of IHCs and OHCs.^[^ ^154^, ^211^ ^]^ In adult mice, trans‐RWM injection can damage hearing more easily than injections performed prior to postnatal day 5.^[^ ^207^, ^210^, ^212^ ^]^ Although some teams reported no change in the ABR threshold in adult mice after the trans‐RWM injection, such ideal outcomes rely heavily on the skill of the surgeon and the use of fine instruments, such as a fine glass needle and a smooth microinjection system.^[^ ^213^, ^214^ ^]^ Furthermore, the trans‐RWM injection combined with the canal fenestration (RWM+CF) technique was recently developed. This method enables the transduction of sensory hair cells with high efficiency while simultaneously reducing injection‐associated damage.^[^ ^131^, ^213^ ^]^ In addition to the trans‐RWM approach, other studies have developed an onto‐RWM approach. Briefly, after incubation with collagenase or hyaluronic acid, a virus‐loaded gel is applied to the RWM.^[^ ^215^, ^216^, ^217^ ^]^ This method may be the least traumatic approach for virus delivery into the inner ear. In humans, the round window membrane (RWM) is easier to access via a transcanal route. Trans‐RWM insertion of an artificial cochlea is a well‐established surgery. Recent clinical trials of AAV‐mediated inner ear gene therapy have adopted the trans‐RWM route and achieved promising outcomes.^[^ ^28^, ^29^, ^30^ ^]^

The canalostomy approach, which includes the posterior semicircular canal and lateral semicircular canal, has been developed in recent years. This method directs the surgical route away from the organ of Corti, and prior studies have reported minimal hearing damage.^[^ ^218^, ^219^, ^220^, ^221^ ^]^ Studies have shown that delivering 1 µL of AAV via the canalostomy approach can preserve hearing function while achieving sufficient infection efficiency in the organ of Corti.^[^ ^136^, ^142^, ^222^ ^]^ However, previous studies have also reported that this approach mainly infects the vestibular system.^[^ ^218^, ^219^, ^220^ ^]^ Additionally, due to the lack of markers, distinguishing whether the needle is in the endolymph or perilymph spaces during injection is difficult.^[^ ^220^ ^]^ In humans, the temporal bone is thick, and drilling into the semicircular canal can easily disturb vestibular function, making this approach less ideal.^[^ ^223^ ^]^

The cochleostomy approach enters either the scala tympani or the scala media more directly. Similar to the approaches mentioned above, early postnatal cochleostomy is less damaging to the auditory brainstem response threshold.^[^ ^210^, ^212^ ^]^ In contrast, opening an ossified cochlea in adult mice always results in a greater risk of irreversible hearing damage.^[^ ^224^, ^225^ ^]^ Due to this significant drawback, cochleostomy is less frequently adopted than the RWM approach and canalostomy approach.

Other systemic approaches, such as intravenous injection through the superior temporal vein, intracerebroventricular injection, and cisterna magna injection, also achieve success to some extent.^[^ ^226^, ^227^, ^228^ ^]^ These systemic approaches avoid the risk of direct surgical damage to the inner ear. However, they may require a relatively high titer of the virus and can have off‐target effects on other organs.^[^ ^229^ ^]^ In addition, anatomical differences between species may affect the therapeutic outcomes. Recently, Mathiesen et al. reported a cerebrospinal fluid conduit approach for AAV delivery in the inner ear of adult mice, which relies on the widely patent cochlear aqueduct in rodents.^[^ ^230^ ^]^ However, little is known about its patency for AAVs in primates/humans, which requires further exploration.^[^ ^227^, ^231^, ^232^, ^233^ ^]^

Each of these methods presents unique challenges and risks, requiring a careful consideration of their application. Increased infection efficiency must be balanced with minimal injection‐associated damage to achieve better AAV delivery outcomes.

These recent developments in AAV capsid and promoter, along with advancements in surgical approaches, have increased the effectiveness and safety of AAV delivery in the laboratory. However, more data from recent clinical trials are needed to determine the optimal surgical approaches, AAV capsids, and promoters. Future aims of AAV‐mediated inner therapy should also focus on enhancing cell‐type specificity and transgene expression stability.

## 4 Disadvantages and Challenges of AAV in Inner Ear Applications

### 4.1 Packaging Size Limitations

One of the well‐known limitations shared among all AAV serotypes is their limited packaging capacity.^[^ ^234^, ^235^ ^]^ The optimal size of the transgene that can be engineered, including regulatory elements, is less than 4.7 kb. Oversized transgenes often result in decreased packaging efficiencies,^[^ ^236^, ^237^ ^]^ significantly impeding the application of AAVs in transgenic therapies for polygenic diseases and even monogenic diseases caused by variants in larger genes.^[^ ^236^, ^238^ ^]^ Additionally, this size limitation constrains the adjustment of regulatory elements, such as promoters and reporter elements.

Various strategies have been employed to address this limitation. One straightforward approach involves reconstructing a smaller version of the target gene while maintaining its primary function. For example, in gene therapy for Duchenne muscular dystrophy, the original dystrophin gene, which is 11.5 kb in length, exceeds the maximum packaging capacity of a single AAV vector. Researchers have therefore developed several truncated forms of dystrophin that can be accommodated within a single AAV vector.^[^ ^239^ ^]^ However, developing a mini version of a target gene is largely case‐specific and can only be achieved based on a thorough understanding of the structure–function relationship of the target gene.^[^ ^38^ ^]^

Another strategy to deliver oversized transgenes is through splitting the transgene into segments, which then combine into a full‐size transgene or functional protein once delivered into the same cell through multiple AAV vectors. Based on the strategies adopted for segment recombination at various stages, they can be briefly divided into DNA, RNA, and protein levels. Below, we introduce the mechanisms of these strategies and their applications.

At the DNA level, transgene recombination through multiple AAVs includes DNA trans‐splicing, overlapping, and a hybrid method. These methods were originally designed to deliver large genes by dividing transgenes into parts and carrying them through two or more AAV vectors. Once the vectors infect the same cell, these transgene pieces undergo head‐to‐tail intermolecular concatamerization to form an intact gene of interest.^[^ ^240^, ^241^ ^]^

#### 4.1.1 DNA Trans‐Splicing Method

Early studies showed that the ITR region alone can serve as a recombination signal with low efficiency.^[^ ^242^, ^243^ ^]^ To overcome this shortcoming, researchers developed engineered splicing signals (including splice donor and splice acceptor) to facilitate the removal of the ITR region and promote recombination efficiency.^[^ ^244^, ^245^, ^246^ ^]^ This procedure produces a full‐length transgene and enables the production of full‐size mRNA, finally generating a full‐size mature protein.^[^ ^241^ ^]^ The process requires precise coordination between the two AAV vectors, and the selection of split sites can be inflexible. This adds complexity to the design and implementation of the method.^[^ ^247^ ^]^

#### 4.1.2 DNA Overlapping Method

This method requires a common sequence shared between two split transgene segments (at the 3' end of the head sequence and the 5' end of the tail sequence).^[^ ^248^, ^249^ ^]^ During co‐transfection, overlapping sequences undergo homologous recombination and mismatch repair to generate an intact full‐length transgene, and subsequent mRNA and protein.^[^ ^238^, ^250^ ^]^ The longer the overlapping region, the higher the recombination efficiency; however, it decreases the packaging capacity.^[^ ^251^, ^252^, ^253^ ^]^ Moreover, longer overlapping sequences can more easily generate secondary structures and decrease recombination efficiency.^[^ ^254^ ^]^ Besides, the selection of the homology sequence has a large impact on the efficiency of transgene recombination and needs to be optimized for different genes.^[^ ^255^ ^]^

#### 4.1.3 DNA Hybrid Method

First proposed by Gosh et al. in 2008, this method combines trans‐splicing and overlapping.^[^ ^256^, ^257^ ^]^ Two homologous sequences are placed after the splice donor in the 5' half of the transgene and in front of the splice acceptor in the 3' half of the transgene, respectively. The full‐size transgene is reconstructed with higher efficiency through both mechanisms.^[^ ^258^, ^259^ ^]^ In this method, the homologous sequences can direct the correct orientation of the transgene recombination.^[^ ^258^ ^]^ Researchers found that using bridging sequences with higher homologous recombination abilities significantly promotes the generation of full‐size transgenes.^[^ ^255^ ^]^ Hence, sequences such as alkaline phosphatase (AP), F1 phage genome (AK), and their minimal fragments were developed to improve transgene expression efficiency.^[^ ^92^, ^258^, ^260^ ^]^ The use of these universal bridging sequences also makes it easier to design the splicing strategy with a less time‐consuming process.

Although DNA trans‐splicing was reported to have relatively low reconstitution efficiency,^[^ ^253^ ^]^ recent research mostly adopts DNA‐level gene recombination to deliver large transgenes into the inner ear. In 2019, Al‐Moyed et al. compared the efficiency of trans‐splicing and the hybrid method in delivering the otoferlin transgene into IHC.^[^ ^261^ ^]^ Their results showed similar otoferlin delivery efficiency and similar functional recovery in *Otof* knockout mice between the two methods. Akil et al. also used the hybrid method to deliver otoferlin, with the alkaline phosphatase sequence used as a bridging sequence to enhance homologous recombination efficiency.^[^ ^92^ ^]^ In 2024, Ivanchenko et al. used the hybrid method to deliver PCDH15 in a mouse USH1F model, successfully restoring hearing and balance in mice.^[^ ^262^ ^]^ The most recent clinical trial of OTOF delivery in patients with DFNB9 deafness also adopted the hybrid method and achieved promising outcomes.^[^ ^28^, ^30^, ^263^ ^]^

#### 4.1.4 mRNA Trans‐Splicing

Developed in 2003 by Pergolizzi et al.,^[^ ^264^ ^]^ mRNA trans‐splicing differs from DNA trans‐splicing as it occurs between two pre‐mRNAs with 5' and 3' splice signals. Full‐size mRNA is generated after such trans‐splicing, enabling further translation into a full‐size functional protein. However, mRNA trans‐splicing is a relatively unexplored method. In 2009, Song et al. achieved the delivery of the cystic fibrosis transmembrane conductance regulator in vitro via the mRNA trans‐splicing method.^[^ ^265^ ^]^ In 2023, Riedmayr et al. further developed it into reconstitution via mRNA trans‐splicing (REVeRT).^[^ ^266^ ^]^ It is believed that REVeRT is more flexible in split site selection. There is still no mRNA trans‐splicing application in the inner ear, which requires further study to define its efficiency and clarify its mechanisms.

#### 4.1.5 Protein Trans‐Splicing

Unlike genetic rearrangement that happens at the DNA or RNA level, this method involves post‐transcriptional linkage between transgene product segments to generate full‐size protein. In the early 1990s, intervening protein segments (inteins) were found in *Saccharomyces cerevisiae*.^[^ ^267^, ^268^ ^]^ These showed the self‐cleaving ability to join two protein segments via peptide bonds.^[^ ^269^ ^]^ For dual AAV‐mediated delivery of large transgenes, the intein is divided into two parts and linked to the 3' end and 5' end of the transgenic protein segments. Once translated into one cell, these parts of inteins undergo join‐cleavage, leading to the combination of the N‐ and C‐terminal parts of protein segments.^[^ ^270^ ^]^ However, this method requires a Cys, Thr, or Ser residue to exist in the 3' half of the first position and correct post‐translational protein folding of both segments.^[^ ^271^, ^272^, ^273^ ^]^ A recent study in retina gene delivery showed that protein trans‐splicing has higher efficiency compared to other dual‐AAV approaches.^[^ ^274^ ^]^ In 2022, Tang and colleagues adopted the dual‐AAV‐mediated protein trans‐splicing method to deliver otoferlin in DFNB9 mice. Their results showed significant hearing functional recovery through this strategy.^[^ ^275^ ^]^ One of the limitations of protein trans‐splicing is that intein should be inserted in loop structures, and finding suitable insertion locations for intein requires extra effort in this strategy.^[^ ^276^, ^277^ ^]^

There is no doubt that developing these strategies broadens the choices for large transgene delivery into the inner ear. However, at present, there is no study systematically comparing the efficacy of these methods in delivering large genes into the inner ear. There is also a lack of evidence to show the long‐term safety of these methods in the treatment of inner ear diseases, which requires further evaluation. Charles Askew and Wade Chien summarized the cDNA size of genes that cause hereditary HL in their review article. They concluded that 78% of these hereditary HL‐associated genes are suitable for single AAV packaging and that 94% can be packaged through the dual AAV method.^[^ ^278^ ^]^ Understanding the potential for generating undesired products, including incomplete or aberrant gene products and isolated regulatory elements resulting from random splicing, is essential. These products may compromise transduction efficiency and hinder definitive characterization.^[^ ^279^, ^280^ ^]^ Methods such as the utilization of nonhomologous ITRs or hybrid‐AAV vector systems that facilitate directional intermolecular recombination of dual AAV genomes, which enables a more precise selection of split sites, have been developed to address these challenges.^[^ ^242^, ^266^, ^281^ ^]^ Additionally, the ratio of the titers of the two AAV vectors is crucial for transduction efficiency in dual AAV systems, with a 1:1 ratio optimizing transduction efficiency.^[^ ^282^ ^]^ The successful delivery of large genes relies on the spontaneous infection of these trans‐splicing units into target cells and the efficiency of their recombination. Increased splitting of the transgene cDNA can make intact recombination more challenging.^[^ ^257^, ^283^ ^]^ In 2023, Hidekane Yoshimura et al. adopted a triple AAV vector system for inner ear gene therapy. However, their results revealed that the spontaneous transduction efficiency of three vectors in the same IHC was up to only 5.9%.^[^ ^284^ ^]^

Other viral vectors, such as adenovirus and lentivirus, have relatively high packaging capacities of ≈8.5 and 9 kb, respectively.^[^ ^285^, ^286^ ^]^ Adenovirus vectors were reported to be able to transduce several cell subtypes of the mammalian inner ear.^[^ ^42^ ^]^ However, adenovirus vectors can provoke a strong immune response, which can lead to inflammation and damage hearing.^[^ ^287^, ^288^ ^]^ The integration of lentiviral vectors into the host genome also raises safety concerns, particularly for long‐term applications.^[^ ^289^ ^]^ These shortcomings, together with their transduction efficiency compared to that of AAVs, hindering their applications.

Overall, the limited packaging capacity of AAV is one of the biggest challenges for large gene delivery in the inner ear. Extending the cargo space in a single AAV particle is nearly impossible. While the dual‐AAV approach increases the overall transgene size limit, it has lower expression efficiency compared to the single‐AAV approach. This limitation can reduce the therapeutic outcome, especially for target proteins with high turnover rates. One potential solution to overcome the limited packaging capacity of AAV in the future is to correct the mutated gene, rather than delivering the full target gene into the inner ear.

### 4.2 Targeting Specificity

One of the limitations shared by AAV‐mediated gene therapies is target cell specificity. Ideally, AAVs should infect target cells as much as possible while showing limited infection in surrounding healthy cells.^[^ ^290^ ^]^ However, targeting specific cell types in AAV‐mediated inner‐ear gene therapy remains a challenge. For example, in hair cell regeneration studies, researchers have focused on manipulating SCs with hair cell differentiation potential (e.g., Lgr5‐positive cells).^[^ ^172^, ^291^, ^292^ ^]^ When correcting mutations that primarily affect sensory hair cells, off‐target delivery of AAV may increase the risk to inner ear homeostasis. Cell‐type‐specific promoters have been recently adopted to limit cell‐type off‐target effects. For example, the Myo15 promoter is specific to hair cells in the inner ear, significantly minimizing transgene expression in other cell types of the inner ear and central nervous system (CNS).^[^ ^138^ ^]^ Similarly, the glial fibrillary acidic protein (GFAP) promoter has been used for gene expression in astrocytes.^[^ ^196^ ^]^ In the cochlea, the GFAP protein is expressed in SCs but not in sensory hair cells, making GFAP an ideal promoter for targeting gene expression in SCs.^[^ ^197^ ^]^

We anticipate that more cell‐type‐specific AAV capsids and promoters will be developed and applied in the future to limit off‐target effects.

### 4.3 Neutralizing Antibodies

Although AAV infection does not appear to pose a threat to the health of animals or humans, increased titers of serum anti‐AAV NAbs, which are triggered primarily by capsids in a dose‐dependent manner, are prevalent in most subjects.^[^ ^293^ ^]^ These neutralizing anti‐AAV immunoglobulin G (IgG) antibodies, resulting from the host humoral immune response following natural AAV infection or systemic administration of AAV vectors,^[^ ^294^ ^]^ are highly prevalent, as they are found in from 30% to 80% of both healthy individuals and patients receiving AAV‐mediated gene therapy.^[^ ^295^, ^296^ ^]^ NAbs can persist for long periods, leading to a higher prevalence of these antibodies in older individuals. In addition to age, differences in NAb prevalence have been observed based on sex, race, geographic location, disease state, and AAV serotype.^[^ ^295^ ^]^

NAbs exert a cross‐reactive neutralizing effect on different AAV serotypes due to the high homology of their capsids, with anti‐AAV2 NAbs exhibiting the highest prevalence among all serotypes. In animal models and human trials, the presence of NAbs has been confirmed to reduce the systemic AAV transduction efficiency by reducing the effective concentration of AAV, shortening the transduction or expression duration and altering the AAV biodistribution.^[^ ^297^ ^]^ This side effect is particularly notable when AAV vectors are delivered to target cells such as hepatocytes, blood cells, and cells of the CNS, which require blood or cerebrospinal fluid translocation.^[^ ^55^, ^298^, ^299^, ^300^ ^]^ Furthermore, significant increases in NAb titers and prevalence following the readministration of AAV vectors have been reported, significantly impeding the efficacy of AAV vectors in subsequent applications.^[^ ^295^ ^]^ Researchers have often minimized the effect of NAbs on AAV following systemic administration by administering immunosuppressants or changing to a serotype different from that used in the first injection.^[^ ^301^, ^302^, ^303^ ^]^ Compared with other organs, the inner ear is regarded as an immune‐privileged organ because of the presence of the BLB.^[^ ^304^ ^]^

Addressing the increase in NAb titers following AAV vector administration remains a crucial challenge in increasing therapeutic efficiency. Previously, some findings also suggested that the increase in NAb titers following AAV administration does not necessarily correlate with adverse safety or efficacy outcomes in the treatment of hemophilia A.^[^ ^305^, ^306^ ^]^

Theoretically, the best way to address the challenge of pre‐existing NAbs is to mutate the AAV capsid to prevent NAb binding. However, the high cross‐reactivity among AAV serotypes makes this goal difficult to achieve.^[^ ^296^, ^307^, ^308^ ^]^ Plasmapheresis, which removes all immunoglobulins, has shown promise in depleting most NAbs from patients’ sera, but only in those with low NAb titers.^[^ ^309^ ^]^ However, this approach has clear drawbacks. Researchers recently reported that NAbs can be removed in vitro by incubating intravenous immunoglobulin (IVIG) or human sera with beads covalently coupled to AAV particles to minimize overall immunosuppression.^[^ ^310^, ^311^ ^]^ Additionally, treating IVIG with imlifidase, a streptococcal cysteine protease that cleaves IgG into F(ab')2 fragments and Fc, was reported to completely digest total IgG and anti‐AAV8 IgG within 24 h after administration.^[^ ^312^ ^]^ This approach enables the transduction of NHPs with pre‐existing neutralizing antibodies and allows the readministration of the same AAV serotype.^[^ ^312^ ^]^ Although these promising approaches require further verification for clinical safety, they offer alternative options for future AAV‐mediated inner ear gene therapy, particularly for patients with pre‐existing NAbs and those needing readministration.

For current clinical trials in the inner ear, there is still limited knowledge about the effect of pre‐existing NAbs on localized AAV injection into the inner ear. It remains to be decided whether patients with pre‐existing NAbs should be excluded from AAV‐mediated inner ear therapy.

### 4.4 Potential Integration of AAVs Into the Genome

AAV genomes primarily persist in the cell nucleus as episomes.^[^ ^86^ ^]^ Wildtype AAVs rarely integrate into the host genome, with in vitro studies showing that only ≈0.1% of wtAAV2 genomes integrate into a specific region (the AAVS1 site).^[^ ^313^ ^]^ Integration into the host genome can lead to more stable and prolonged expression of the AAV genome.^[^ ^314^ ^]^ However, such a low integration rate makes it less likely to be advantageous. Additionally, since this integration is nonrandom, it poses less of a risk of tumorigenesis.^[^ ^313^ ^]^ Moreover, recombinant AAV has the rep gene removed, which significantly reduces genome integration.^[^ ^38^ ^]^ In comparison, retroviruses integrate proviral DNA into the chromosomal DNA of their host at many different sites. Most integrations are benign, but some result in cancer.^[^ ^315^, ^316^ ^]^ Due to the low integration rate of AAVs and the relatively low dose applied locally in the inner ear, AAVs are considered safer vectors. However, long‐term follow‐up observations are still needed to improve the safety of AAV‐based transgenic therapy.

### 4.5 Risks of AAV Systemic Administration

Following systemic AAV administration, some cases of organ dysfunction have been reported.^[^ ^317^, ^318^, ^319^ ^]^ Specifically, liver function abnormalities have been observed in patients receiving AAV‐mediated gene therapy, particularly those with hemophilia, and these abnormalities may persist for a prolonged period.^[^ ^320^ ^]^ Notably, two patients with X‐linked myotubular myopathy died after gene therapy with a high dose of AAV due to hepatotoxicity.^[^ ^321^ ^]^ According to the current literature, hepatotoxicity may be attributed to an increase in Nab titers, which triggers hepatocyte necrosis, whereas elevated alanine aminotransferase (ALT) levels may be associated with cytotoxic T‐cell responses to the AAV capsid.^[^ ^60^ ^]^ However, the precise relationship between hepatotoxicity and AAV administration remains unclear. Additionally, renal failure has been reported in several patients participating in different gene therapy clinical trials and is often accompanied by complement disorders.^[^ ^239^, ^321^ ^]^ Further research is needed to elucidate the mechanisms underlying these adverse effects.

Compared with systemic administration, local injection of AAV into the cochlear perilymph and/or endolymph is expected to reduce the leakage of AAV into the circulatory system.^[^ ^322^ ^]^ However, recent clinical trials reported detectable levels of the AAV DNA on the first day following RWM injection, which became undetectable three days after the injection.^[^ ^30^ ^]^ The implications of this transient leakage remain unclear, and whether leakage can lead to systemic side effects remains uncertain; therefore, further investigations are needed. Additionally, exploring strategies to mitigate this leakage is crucial, such as reducing the injection volume while simultaneously increasing the concentration of the AAV vector.

## 5 Strategies and Application Scenarios of AAV in Hearing Loss

### 5.1 Different AAV‐Based Strategies for Inner Ear Therapy

Through years of development in various fields, several AAV‐mediated gene therapy strategies have matured. Researchers have adopted these strategies to treat inner ear diseases and have achieved promising outcomes in preclinical and clinical trials.^[^ ^323^ ^]^ AAV‐mediated gene therapy for inner ear diseases can involve adding, removing, or altering the genetic material in targeted cells in the cochlea to achieve a therapeutic effect.^[^ ^26^, ^27^ ^]^ Understanding the pathological mechanisms of different types of inner ear diseases is essential before effective AAV‐mediated therapy can be successful. Below, we have summarized three main strategies that have been developed in recent years.

#### 5.1.1 Gene Replacement

One simple way to achieve a therapeutic effect is by directly expressing (or overexpressing) specific proteins to support the normal function of target cells, to trigger defensive effects, or to induce repair mechanisms to achieve hearing protection.^[^ ^38^, ^324^ ^]^ Specifically, when the original functional gene is disrupted or insufficient, AAV can carry exogenous cDNA to the target cell, thereby achieving a therapeutic effect.^[^ ^325^ ^]^ This strategy is relatively straightforward; however, it can be hindered by the size of the target gene.^[^ ^235^, ^326^ ^]^ The dual‐AAV approach or the use of a mini version of the target cDNA are alternative solutions for large gene therapy.^[^ ^266^, ^327^ ^]^ In addition, long‐term treatment effects can be achieved only when the exogenous gene used for treatment can be expressed continuously.^[^ ^314^, ^328^ ^]^ As mentioned above, although not integrated into the host genome in most cases, AAVs can express exogenous genes steadily, which contributes to a stable treatment effect.^[^ ^90^ ^]^

#### 5.1.2 Gene Silencing

In contrast to the direct expression of exogenous genes, another method involves removing or silencing a “harmful gene” or a gene that induces damaging effects due to mutation or inner ear trauma. Before AAVs became popular for gene silencing, one of the most common tools for gene silencing was RNA interference (RNAi). These short sequences dsRNA (≈21–22 bp) have characteristic 2 nt 3′ overhangs, enabling recognition by the RNAi enzymatic machinery.^[^ ^329^, ^330^ ^]^ This recognition ultimately leads to the homology‐dependent degradation of the target mRNA.^[^ ^331^ ^]^ Although the direct delivery of a small interfering RNA (siRNA) may achieve temporary target gene silencing and can be adopted for some functional studies, the main shortcoming is a narrow treatment window, which limits the application of siRNAs in most cases of chronic and hereditary HL.^[^ ^332^, ^333^, ^334^, ^335^, ^336^ ^]^ shRNAs were developed to overcome this limitation. They consist of two complementary 19–22 bp RNA sequences linked by a short loop of 4–11 nt, resembling a hairpin.^[^ ^337^, ^338^ ^]^ The mechanism of shRNA in gene silencing is similar to that of siRNAs, with the main difference being that shRNAs can integrate into the AAV vector. After transcription, the shRNA sequence is exported to the cytosol, where it is recognized by the enzyme Dicer, which processes it into siRNA duplexes.^[^ ^339^ ^]^ Compared with the use of siRNAs, the packaging shRNA into an AAV expression vector allows for a longer treatment window.^[^ ^340^ ^]^ In addition to RNA interference, gene silencing can also be achieved through CRISPR‐based gene disruption, which theoretically can provide a longer‐lasting treatment effect.^[^ ^341^, ^342^ ^]^ One of the challenges for AAVs to carry the CRISPR system is its limited packaging capacity, making it difficult for the full SpCas9 (Cas9 enzyme from *Staphylococcus pyogenes*) enzyme sequence (4.2 kb), the sgRNA sequence, and associated promoters and regulatory sequences such as polyA ends to be inserted into one vector.^[^ ^238^, ^343^ ^]^ Researchers developed a strategy by splitting the expression of Cas9‐intein halves into two vectors, with homologous‐directed repair (HDR) occurring during the expression of each part, finally creating a functional Cas9 protein.^[^ ^344^ ^]^ In addition, a novel Cas9 from *Staphylococcus aureus* (SaCas9) was found to be smaller (≈3.2 kb) than traditional SpCas9, enabling the editing enzyme, sgRNA, and regulatory elements to be packaged in a single viral particle.^[^ ^345^, ^346^ ^]^ CRISPR‐based gene silencing can specifically target and silence cDNA or mRNA with the guidance of a small guide RNA (sgRNA).^[^ ^347^, ^348^, ^349^ ^]^ Although challenges such as off‐target effects persist,^[^ ^350^, ^351^ ^]^ recent developments in CRISPR techniques and AAV vectors have allowed researchers to specifically and continuously silence target genes in the inner ear.

#### 5.1.3 Gene Correction

In contrast to the full removal or insertion of the target gene, gene correction represents a more precise healing method for the problem gene. CRISPR‐based gene correction is a next‐generation gene therapy that has developed rapidly in recent years.^[^ ^352^, ^353^, ^354^, ^355^ ^]^ The specificity of the CRISPR gene correction system also relies on the design of the sgRNA and protospacer adjacent motif (PAM).^[^ ^355^, ^356^, ^357^, ^358^, ^359^ ^]^ This revolutionary technology simplifies the complex protein design and engineering work into the much easier task of designing an ideal guide RNA sequence for the genomic target site.^[^ ^360^ ^]^ A regular CRISPR gene correction procedure includes the deletion of the target base or sequence and the repairing the DNA break through HDR. However, studies have shown that HDR‐based knock‐in has relatively low efficiency.^[^ ^361^, ^362^ ^]^ Methods such as nonhomologous end joining (NHEJ), microhomology‐mediated end joining (MMEJ), and homology‐mediated end joining (HMEJ) were subsequently developed to increase the knock‐in efficiency.^[^ ^363^, ^364^, ^365^ ^]^ In addition to these ‘cut and heal’ methods, ‘base editing’ via the fusion of the Cas9 enzyme (e.g., fusion with cytidine deaminase) is another strategy to correct point mutations more directly. Base editing efficiencies have been found to be greater than those of HDR‐mediated point mutation correction.^[^ ^366^, ^367^ ^]^ Finally, regulating protein expression through Cas13‐based RNA editing is another method that does not involve genome changes but rather alters the mRNA and protein products that are generated.^[^ ^368^ ^]^ This regulation is reversible and provides researchers with more options than permanently changing the genome.^[^ ^369^ ^]^ AAV‐mediated CRISPR‐based gene correction is relatively more stable and efficient than virus‐free methods.^[^ ^370^, ^371^, ^372^ ^]^ Currently, several preclinical trials have shown that AAV‐mediated CRISPR gene correction is a promising method for treating hereditary deafness.

Although current AAV‐based clinical trials for inherent hearing loss mainly adopt a gene replacement strategy, it is possible that gene correction will be applied in the future. Gene correction is one way to overcome the limited packaging capacity of AAV and is an ideal method to remove toxic mutant proteins at the gene level. However, the gene correction strategy also faces challenges such as off‐target effects.

### 5.2 AAV Application Scenarios for Hearing Loss

Hearing loss can be classified by type, onset, severity, and frequency.^[^ ^373^ ^]^ The onset of hearing loss is divided into prelingual (before speech development) and postlingual (after speech development) hearing loss. While all congenital hearing loss is prelingual, not all prelingual hearing loss is congenital.^[^ ^374^, ^375^ ^]^ Hearing loss can also be categorized by cause: genetic or acquired.

Genetic hearing loss may follow autosomal dominant, autosomal recessive, X‐linked Mendelian, or mitochondrial inheritance patterns. Hereditary factors account for more than 50% of congenital hearing impairment cases in developed countries,^[^ ^376^ ^]^ with ≈70% being nonsyndromic and predominantly monogenic.^[^ ^27^ ^]^ The monogenic feature makes this type of HL especially suitable for gene therapy. Severe to profound prelingual deafness is typically associated with autosomal recessive inheritance, whereas progressive, less severe forms appearing from late childhood onward are generally linked to autosomal dominant patterns^[^ ^377^, ^378^ ^]^

Acquired hearing loss results from environmental factors such as noise exposure, ototoxic drugs, infection, and aging, which have been extensively studied in recent years.^[^ ^379^, ^380^, ^381^, ^382^ ^]^ Notably, acquired hearing loss can arise from a combination of genetic factors that interact with environmental factors, leading to pathological changes within the ear that often involve complex mechanisms. Environmental risk factors significantly contribute to acquired hearing loss throughout one's lifespan.^[^ ^383^ ^]^ Within the inner ear, sensory hair cells, especially OHCs, are sensitive to traumatic stimulation.^[^ ^384^, ^385^ ^]^ Two main strategies using AAV to treat acquired hearing loss in preclinical research are AAV‐mediated hair cell regeneration and AAV‐mediated gene manipulation.^[^ ^288^ ^]^

In the past decade, the combination of novel technologies and the development of AAVs has significantly accelerated progress in gene therapy for inner ear diseases. These breakthroughs have paved the way for innovative treatments for conditions once they are deemed untreatable, providing new hope to numerous patients. In this section, we highlight several exemplary cases of gene therapy for hearing loss, tracking the trajectory of these therapies from laboratory research to clinical trials. Below, we categorize these examples of preclinical applications based on their therapeutic strategies and the specific diseases they target.

#### 5.2.1 Genetic Hearing Loss

Gene replacement is suitable for the treatment of inherited recessive HL caused by nonsense mutations or mutations not involving dominant‐negative effects. In other words, the mutation should only cause the loss or insufficient expression of a protein, with the generated mutated protein being nonfunctional or having reduced function.^[^ ^386^ ^]^ One typical example of AAV‐mediated gene replacement is seen in the vesicular glutamate transporter‐3 knockout model (*Vglut3* ^‐/‐^). Normally *Vglut3* is expressed mainly in IHCs and is critical for glutamate‐mediated synaptic transmission.^[^ ^387^, ^388^ ^]^ Moreover, the *Vglut3* cDNA (≈1.8 kb) can be packaged into one AAV vector. Because of these features, AAV‐mediated *Vglut3* replacement has been shown to achieve profound hearing improvements in both neonatal and mature *Vglut3* knockout mouse models.^[^ ^389^, ^390^ ^]^

Another gene that has been well studied for inner ear gene therapy in recent years is *OTOF*, which encodes otoferlin, and it is mainly expressed in IHCs; otoferlin plays a critical role in signal transduction from auditory cells to synapses.^[^ ^391^, ^392^ ^]^ *OTOF* mutations, cause nonsyndromic autosomal‐recessive HL, known as DFNB9, despite a normal sensory epithelium structure.^[^ ^393^, ^394^ ^]^ Clinical data show that *OTOF* mutations can include nonsense mutations, frameshifts, amino acid deletions, and missense mutations.^[^ ^392^ ^]^ *OTOF* mutations account for 2–8% of cases of hereditary deafness.^[^ ^28^, ^395^, ^396^ ^]^ *Otof* knockout mice serve as common laboratory model animals for studying the function of otoferlin and targeted treatment.^[^ ^397^, ^398^ ^]^ *Otof* mutation is an ideal model for AAV‐mediated gene replacement treatment studies since most AAV serotypes have greater infection efficiency in IHCs than in OHCs, especially in mature mice,^[^ ^117^, ^136^ ^]^ and *Otof* mutation, in most cases, is associated with insufficient otoferlin expression. On the other hand, the large otoferlin cDNA (≈6 kb) exceeds the packaging capacity of a single AAV particle, posing a challenge to researchers. In 2019, two independent teams used similar dual‐AAV methods to treat *Otof* ^‐/‐^ mice and both achieved outstanding hearing restoration effects. A homologous recombinogenic bridging sequence was used to combine the N‐terminal and C‐terminal halves of the otoferlin coding sequences.^[^ ^92^, ^261^ ^]^ These promising results have attracted the attention of researchers worldwide.

Transmembrane channel‐like 1 (TMC1) is expressed in both IHCs and OHCs and affects the permeation of sensory transduction channels in cochlear sensory hair cells.^[^ ^399^, ^400^ ^]^ Mice with loss‐of‐function mutations in *Tmc1* exhibit severe autosomal‐recessive HL and balance dysfunction despite normal hair cell morphology.^[^ ^400^ ^]^ In 2015, Charles Askew and colleagues successfully used AAV2/1 carrying *Tmc1* (≈2.3 kb) to restore hearing function in *Tmc1* loss‐of‐function mutant mice (*Tmc1* ^Δ/Δ^).^[^ ^118^ ^]^ Their findings also indicate the recovery of the transduction current in both IHCs and OHCs. Similarly, in 2019, Carl A. Nist‐Lund and colleagues reported that the delivery of TMC1‐ and TMC2‐expressing AAVs to neonatal *Tmc1* ^Δ/Δ^ mice robustly increases hair cell survival and restores hearing and vestibular function.^[^ ^401^ ^]^

In addition to targeting sensory hair cells, researchers have also performed gene replacement studies in other inner ear structures. For example, the *Kcnq1* gene (≈2 kb) is normally expressed in marginal cells in the SV of the inner ear and is responsible for voltage‐gated potassium channel expression. KCNQ1 plays an important role in the secretion of K^+^ into the endolymph to maintain the EP.^[^ ^402^, ^403^ ^]^ Mutations in the *KCNQ1* gene cause recessive HL associated with Jervell and Lange–Nielsen syndrome.^[^ ^404^ ^]^ Common mutations in *Kcnq1* are missense mutations that result in amino acid replacements. Qing Chang et al. successfully performed AAV1‐mediated KCNQ1 replacement in marginal cells in the SV and significantly restored hearing in *Kcnq1* ^‐/‐^ mice.^[^ ^405^ ^]^ They also reported that this exogenous KCNQ1 protein correctly trafficked to its native membrane location.

Other gene replacement strategies have been adopted in similar studies using different hereditary HL animal models, such as *Syne4* ^‐/‐^, *Msrb3* ^‐/‐^, *Slc26a4* ^‐/‐^, *Gjb2* ^cko^, *Tmprss3* ^‐/‐^, *Clrn1* ^‐/‐^, *Tprn*, and *Usher1c* ^c.216G>A^ animals, and have restored hearing or vestibular function to different extents.^[^ ^112^, ^142^, ^406^, ^407^, ^408^, ^409^, ^410^, ^411^, ^412^ ^]^

Notably, the efficacy of gene replacement therapy largely depends on the stable expression of exogenous cDNA in the original cells, which requires this DNA to perform its normal functions. Because of the genome nonintegrating feature of AAV, transgene expression can be lost over time.^[^ ^222^, ^389^ ^]^ AAV readministration may be required to achieve long‐term treatment effects. However, as mentioned above, primary administration can induce the production of NAbs through host immune responses, which can abolish the treatment effect of AAV repeated administrations.^[^ ^413^, ^414^ ^]^ Reducing the generation of NAbs through necessary immunosuppression or switching AAV serotypes may be necessary to increase gene delivery efficiency and long‐term safety following repeated administration.^[^ ^301^, ^415^, ^416^ ^]^ In addition, the overproduction of complementary proteins may not always produce a positive treatment effect, and because of the irreversibility of this process, these proteins cannot be removed once they are delivered to target cells. Exogenous production may exceed the turnover ability of sensory hair cells and induce damaging effects. Therefore, an expression control system may be considered to make gene modification safe and efficient.^[^ ^386^ ^]^

Gene disruption is suitable for treating autosomal dominant hearing loss. Autosomal dominant hearing loss manifests when a single dominant allele within the responsible gene is sufficient to express the phenotype.^[^ ^417^ ^]^ Consequently, in most cases, individuals diagnosed with autosomal‐dominant nonsyndromic HL have at least one hearing‐impaired parent.^[^ ^418^, ^419^ ^]^ Affected individuals generally have bilateral HL, which develops after the ability to speak, is progressive, and varies in severity from mild to profound.^[^ ^420^ ^]^ To date, over 50 genes related to autosomal dominant nonsyndromic HL have been identified.^[^ ^417^ ^]^ The genomic features of autosomal dominant nonsyndromic HL make it suitable for vector‐mediated gene disruption therapy.

One of the well‐studied models of DFNA36‐associated HL is the Beethoven (Bth) mutation mouse model. *Bth*/+ mice carry an orthologous missense mutation (c.T1235A, p.M412K) in the *Tmc1* gene and exhibit progressive hair cell loss starting at one month of age.^[^ ^421^ ^]^ This mutation results in dominant HL, making gene replacement strategies unsuitable for this disease. In 2018, Xue Gao and colleagues applied lipid‐based Cas9‐mediated gene disruption to treat *Bth*/+ mice. Their results indicated significant hearing function preservation with no detectable off‐target effects.^[^ ^370^ ^]^ Based on their promising findings, many teams have attempted to use AAV‐mediated gene disruption strategies in the same model. For example, in 2019, Hidekane Yoshimura and colleagues performed AAV‐mediated RNAi‐mediated gene silencing in *Bth*/+ mice. This treatment slowed the progression of HL and increased IHC survival in the mature *Bth*/+ model mice within a temporal window for successful intervention of between 8 and 12 weeks after birth.^[^ ^422^ ^]^ As mentioned above, in the Cas9 method, sgRNA is utilized to target DNA. Due to the mismatch tolerance between the sgRNA and the target DNA sequence, the off‐target effect of the traditional sgRNA strategy is nonnegligible. Recently, studies have indicated that the PAM sequence itself can distinguish mutant alleles from WT alleles with very limited tolerance.^[^ ^423^, ^424^ ^]^ Bence György et al. adopted AAV‐mediated SaCas9‐KKH to disrupt the mutant allele selectively and efficiently in *Bth*/+ mice.^[^ ^91^ ^]^ The use of SaCas9‐KKH‐based gene disruption through AAV‐Anc80L65 showed high specificity for the *Bth* allele, with no detectable cleavage at any genome‐wide off‐target sites. Importantly, the hearing preservation effect lasted for more than 24 weeks, which is more durable than that of the lipid‐based method. Recently, a CRISPR‐mediated gene silencing strategy called CasRx was developed to target posttranscriptional mRNAs. Unlike methods that permanently alter the genome, the effectiveness of CasRx relies on the RNA knockdown efficiency.^[^ ^425^, ^426^, ^427^ ^]^ It selectively and efficiently reduces the expression of both coding and noncoding RNAs, outperforming short‐hairpin‐RNA‐based interference.^[^ ^428^ ^]^ In 2022, Ziwen Zheng et al. reported the use of the CasRx‐based mRNA interruption method to prevent hearing loss in *Bth*/+ mice.^[^ ^429^ ^]^

Another example of AAV‐mediated gene disruption therapy for inner ear diseases involves experiments with the *Myo6* semidominant mouse model. MYO6 gene mutations can cause either autosomal dominant inherited HL (DFNA22) or autosomal‐recessive inherited HL (DFNB37).^[^ ^430^, ^431^ ^]^ In mice, the p.C442Y mutation in *Myo6* leads to semidominant hereditary HL and profound sensorineural HL by middle age.^[^ ^432^ ^]^ In 2022, Yuanyuan Xue et al. reported AAV.PHP.eB‐mediated SaCas9‐KKH‐based mutant gene disruption in *Myo6* ^WT/C442Y^ mice.^[^ ^433^ ^]^ In their design, the mutant site was also covered by the PAM sequence, and the cleavage efficiency and specificity were high, with limited off‐target effects on the WT allele. Inspiringly, the hearing protection effect of this method lasted for more than 5 months. Their teams also achieved success in the Atoh1‐GFP; *Kcnq4* ^WT/G229D^ mouse model using a similar approach, with an editing efficiency in sensory hair cells of ≈34%.^[^ ^434^ ^]^

Compared with gene replacement, in the AAV‐mediated gene disruption strategy for treating autosomal‐dominant HL, the design of an accurate sgRNA sequence and the PAM recognition site of the Cas9 enzyme to differentiate the mutant allele from the WT allele is needed.^[^ ^435^, ^436^ ^]^ The treatment outcomes always depend on the specificity and efficacy of the selected sequences. The spectrum of autosomal dominant HL diseases that can be treated through gene disruption will expand with the development of novel or artificially engineered Cas9 proteins. Although AAV‐mediated gene replacement has already advanced to clinical trials, we believe that the gene disruption strategy will be used in clinical applications in the coming years.

Gene correction is the direct removal of mutations. One ideal approach to treat not only autosomal dominant but also autosomal recessive HL is to specifically correct the mutated allele to the WT sequence. In recent years, the development of universal CRISPR‐mediated base editor enzymes has made such a strategy feasible.^[^ ^437^ ^]^ The toolkit of both DNA and RNA base‐editing enzymes has largely expanded to enable C > T, A > G, C > G, A > I, and C > U conversions.^[^ ^366^, ^367^, ^438^, ^439^, ^440^ ^]^ Moreover, base editing can lead to base corrections in both dividing and nondividing cells, providing an advantage over HDR‐based gene correction, which is limited to dividing cells and necessitates the delivery of a donor template.^[^ ^441^ ^]^ A clinical trial of a therapy involving base editing for sickle cell disease began last year.^[^ ^442^ ^]^ Since mammalian sensory hair cells are nondividing cells, several teams have recently attempted AAV‐mediated base editing to correct point mutations in mammalian models of hereditary HL.

PCDH15 is expressed mainly in the stereocilia of sensory hair cells and is important for initiating the electrical response to sound.^[^ ^443^ ^]^ R245X is a common mutation that occurs in individuals with type 1 Usher syndrome; this mutation involves a change in the Arg245 codon to a stop codon in the Pcdh15 mRNA and likely results in nonsense decay of the PCDH15 protein.^[^ ^444^ ^]^ Last year, Cole W. Peters and colleagues reported the use of base editing to restore hearing in *Pcdh15* ^R245X/R245X^ mice.^[^ ^445^ ^]^ These researchers used dual‐AAV‐packaged adenosine base editors (ABEs) to correct the C > T mutation in humanized *Pcdh15* ^R245X^ mice. Their results revealed that the hearing restoration effect persisted until postnatal day 80. Another mouse model of human nonsyndromic deafness (DFNB23), *Pcdh15* ^av‐3j^, involves the insertion of a single adenine (A) nucleobase, which causes a frameshift in the 7.9 kb Pcdh15 transcript. Homozygous av3j mice develop profound congenital deafness and vestibular dysfunction due to mechanotransduction channel function disorders.^[^ ^443^ ^]^ In 2022, Lian Liu and colleagues designed an m‐3j‐gRNA that was able to reverse the frameshift.^[^ ^446^ ^]^ However, such frameshift corrections did not precisely remove the inserted A but instead deleted 1 (83.9%) or 4 (5.7%) bases or inserted 2 bases (5.5%) near the insertion site. After this sequence was injected into av3j/av3j; Cas9+ mice, hearing and balance were partially restored.^[^ ^446^ ^]^

In addition to DNA base editing, researchers have also performed RNA base editing to correct point mutations in recent hereditary HL studies. For example, in 2022, Qingquan Xiao et al. used AAV‐mediated mini dCas13X.1‐based adenosine base editor (mxABE) delivery to correct the G > A point mutation in *Myo6* ^C442Y/+^ mice. With this method, hearing was restored in mice for 3 months.^[^ ^447^ ^]^ In 2023, this same team introduced an enhanced mini‐dCas13X RNA base editor (emxABE) delivered via AAV to restore hearing in humanized *Otof* ^Q829X/Q829X^ mice.^[^ ^397^ ^]^ Their results revealed an ≈80% A‐to‐I (TAG>TGG) conversion efficiency, and the hearing restoration effect lasted more than 7 months.

Compared with the gene replacement strategy, one main advantage of AAV‐mediated base editing in treating autosomal recessive hereditary HL is that base editing is less limited by the AAV packaging capacity. For large genes such as *Otof* (≈6k bp) and *Pcdh15* (≈5.8 kb), although dual and even triple AAV‐mediated gene replacement can achieve a treatment effect, the split expression efficiency relies on homologous recombination, and more splits may reduce the expression efficiency. Future development of the base editing toolbox may make base editing an ideal solution for correcting autosomal‐recessive hereditary HL, especially forms associated with mutations associated with large genes. In addition, unlike gene disruption, which depends on the normal function of the WT allele, for large‐gene heterozygote haploinsufficiency, homozygote‐associated hereditary HL, and homozygous autosomal dominant hereditary HL, AAV‐mediated base editing may be the ultimate solution.

#### 5.2.2 Acquired Hearing Loss

Hair cell regeneration Mammalian inner ear sensory hair cells are permanent cells with no ability to regenerate or divide once damaged by internal or environmental factors.^[^ ^448^, ^449^ ^]^ Currently, the efficacy of clinical treatments, including hearing aids and CIs, depends on the remaining functional hair cells.^[^ ^450^, ^451^ ^]^ One promising strategy for treating damaged cochleae is to promote the regenerative ability of cochlear progenitor cells, such as SCs, to repair the structure and function of the cochlea.^[^ ^452^, ^453^ ^]^ Owing to its nonpathogenic and long‐term expression features, AAV has been adopted as a candidate vector in studies of hair cell regeneration therapy. Although much more work is needed to transition from laboratory studies to clinical application, some success has been achieved in research on AAV‐mediated hair cell regeneration.^[^ ^169^, ^170^, ^171^, ^172^, ^173^, ^174^, ^292^, ^454^ ^]^

To date, three main strategies have been developed for hair cell regeneration: direct differentiation of SCs to alter their cell type to become functional hair cells, triggering surrounding cells to enter the cell cycle and then differentiate into hair cells, and activating the remaining hair cells to divide to produce new hair cells. SCs are considered excellent candidates for regeneration therapy.^[^ ^455^, ^456^ ^]^ Therefore, unlike most hereditary HL treatments, in which sensory hair cells are directly targeted, hair cell regeneration focuses on SCs, especially those with stemness potential, such as *Lgr5*‐positive SCs.^[^ ^172^, ^173^, ^457^, ^458^, ^459^, ^460^ ^]^ Owing to the capsid reconstruction of AAVs in recent decades, novel serotypes, such as AAV2.7m8, AAV‐ie, and AAV‐ie‐K558R, can infect SCs to different extents.^[^ ^149^, ^160^, ^169^, ^171^ ^]^

Detailed regulation of the complex signaling network associated with cell division, proliferation, and differentiation is needed to promote the regeneration ability of SCs. This process requires the overexpression or silencing of specific genes during precise periods. An outline of the pathway for hair cell regeneration from SCs has been depicted by researchers worldwide.^[^ ^292^ ^]^ In this pathway, the activation of *Atoh1* and *Sox2* and the inhibition of Notch are required for progenitor cells to become presensory cells.^[^ ^461^, ^462^, ^463^, ^464^, ^465^ ^]^ The activation of *Bmp4* and *Fgf2* and the silencing of *Sox2* and *Shh* are necessary for presensory cells to develop hair cell features through differentiation.^[^ ^460^, ^466^, ^467^ ^]^ After differentiation, these native hair cells still require several additional signals, such as *Pou4f3*, *Gfi1*, and *Math1*, to become mature hair cells.^[^ ^468^, ^469^ ^]^ Finally, the induction of *Tbx2* can prompt these mature hair cells to differentiate into IHCs, and the activation of both *Ikzf2* and Insm1 enables the mature hair cells to differentiate into OHCs.^[^ ^470^, ^471^, ^472^ ^]^ Although exciting findings continue to emerge, some molecules important for these proliferation and differentiation processes are still unknown, and further discovery and analysis are needed.

The regeneration of mature functional hair cells necessitates the activation and silencing of various molecules in these complex pathways rather than a single molecule. For example, the overexpression of Atoh1 alone can induce the regeneration of native hair cells in sensory and nonsensory regions, but these new hair cells have rudimentary mechanotransduction features and are not sufficient for normal functioning like mature hair cells.^[^ ^463^, ^473^, ^474^ ^]^ Moreover, persistent overexpression of ATOH1 can result in impaired stereocilia in both endogenous and newly formed hair cells.^[^ ^174^ ^]^ Another example is that inhibition of Notch can induce hair cell regeneration; however, this process is accompanied by the loss of SCs, which in turn leads to the shedding of newly generated hair cells.^[^ ^452^, ^475^ ^]^ Unlike hereditary HL, which can be resolved by regulating a single gene, coregulation of multiple genes is necessary for hair cell regeneration therapy. Experiments with transgenic mice have revealed that the coregulation of multiple molecular components is required to achieve hair cell regeneration, hence providing better answers for regeneration gene therapy. In 2017, Bradley and colleagues reported that the coregulation of *Gata3* or *Pou4f3* and *Atoh1* promoted the conversion of SCs to hair cells in adult mice.^[^ ^476^ ^]^ In 2019, Yan et al. successfully generated mature and functional hair cells through the coexpression of *Gfi1*, *Pou4f3*, and *Atoh1* (GPA) in SCs.^[^ ^468^ ^]^ These findings provide necessary knowledge for the regulation of pathways to treat HL diseases through regeneration methods. However, for future clinical applications, a safe vector is needed to provide these stable manipulation effects. AdV was first adopted for hair cell regeneration research because of its large packaging capacity. AdV‐induced ATOH1 overexpression was used in a clinical trial that began in 2013 (NCT02132130). Although no serious adverse events were observed in 22 participants, slight HL was identified in 32% of the participants. In hearing assessments using pure tone audiometry, no significant improvement in hearing was observed. Based on these results, this trial was suspended in 2023.^[^ ^477^ ^]^ Chen et al. reported that AdV‐mediated overexpression of MATH1 and PAX2 promoted hair cell regeneration after neomycin‐induced damage to the mouse organ of Corti.^[^ ^478^ ^]^ Similarly, Burns et al. delivered *Oct3/4*, *Klf4*, *Sox2*, and *c‐Myc* to cells via AdV‐mediated cotransduction, which significantly stimulated the proliferation of SCs in the mouse utricle.^[^ ^479^ ^]^ Another team reported that the AdV‐mediated temporary activation of *Myc*, *Notch*, and *Atoh1* successfully transformed SCs into hair cells in the adult cochlea.^[^ ^480^ ^]^

As mentioned above, due to the shared features of AdVs, such as their carcinogenic effects and potential to induced an immune reaction, AdV‐mediated hair cell regeneration poses strong risks in future clinical applications.^[^ ^481^, ^482^ ^]^ For clinical translation purposes, AAV‐mediated multigene manipulation to regenerate hair cells has advantages. Recently, researchers used dual‐AAV‐mediated gene cocktails to increase the expression of ATOH1, GFI1, POU4F3, and SIX1 (GPAS) in SCs in vivo and partly achieved hair cell regeneration, with a functional recovery similar to that of native IHCs.^[^ ^170^ ^]^

Several key points need to be addressed before further success can be achieved with AAV‐mediated hair cell regeneration therapy and before this method can be used in clinical applications. Additionally, the risks must be considered. First, the pathway involved in the functional regeneration of hair cells still needs to be clearly elucidated. Second, long‐term overexpression or silencing methods are currently adopted for target gene manipulation in most hair cell regeneration studies. The oncogenic effects should be carefully considered when inserting proliferation‐related genes or silencing cell cycle‐related genes, and subjects should be monitored for these effects.^[^ ^483^, ^484^ ^]^ Finally, engineered AAV serotypes and the development of promoters that specifically target SCs will help to reduce unexpected reprogramming of other cells in the inner ear. Clinical trials designed to address these challenges will need to be performed by researchers worldwide for the clinical translation of AAV‐mediated hair cell regeneration in the future.

#### 5.2.3 Prophylactic Intervention

While the therapeutic window for hair cell regeneration therapy may be wider than that for HL prevention, preventing sensorineural hearing loss (SNHL) may be more realistic than regeneration. Although ongoing clinical trials are being conducted for treating acquired HL, the number of available drugs is still very limited.^[^ ^485^, ^486^, ^487^ ^]^ Recent advancements in AAV‐mediated gene therapy in preclinical experiments have shown promise in preventing acquired HL. Compared with RNAi with a naked siRNA, which has a short half‐life, AAV‐mediated gene therapy is associated with greater stable expression, widening the effective treatment window.^[^ ^335^, ^340^, ^488^ ^]^

Like regeneration strategies, a good understanding of the pathological mechanisms underlying these diseases is needed to manipulate gene expression and treat SNHL. For example, the accumulation of reactive oxygen species (ROS) in sensory hair cells after noise and ototoxic drug exposure is well documented to result in hearing damage.^[^ ^489^, ^490^, ^491^ ^]^ Drugs designed to reduce ROS accumulation by directly abolishing ROS production or indirectly promoting antioxidant activity have shown profound protective effects.^[^ ^492^, ^493^, ^494^, ^495^, ^496^ ^]^ Additionally, calcium overload, energy depletion, and autophagy disruption during exposure to damaging factors have been reported to aggravate hair cell and sensory neuron degeneration.^[^ ^489^, ^497^, ^498^, ^499^ ^]^ These pathways ultimately initiate the apoptotic process in hair cells and sensory neurons.^[^ ^500^ ^]^ Based on these findings, many laboratories have recently adopted AAV‐mediated gene therapy targeting these molecules, and promising results have been reported.

In 2007 and 2015, two independent teams reported that AAV‐mediated X‐linked inhibitor of apoptosis protein (XIAP) overexpression alleviates cisplatin‐induced HL.^[^ ^501^, ^502^ ^]^ In 2021, Wenwen Liu et al. overexpressed the antioxidant enzyme peroxiredoxin 1 (PRDX1) in SGNs through AAV‐Anc80L65, which led to strong protection against cisplatin‐induced HL.^[^ ^503^ ^]^ Xi Gu et al. adopted the SpCas9 gene editing system and AAV transduction to disrupt the Htra2 gene and prevent neomycin‐induced HL.^[^ ^488^ ^]^ Their team also used the Cas13 system to disrupt the *Htra2* mRNA and produce a similar protective effect on neomycin‐induced HL.^[^ ^504^ ^]^ In 2022, our team adopted an AAV2.7m8‐mediated calmodulin‐dependent protein kinase silencing strategy and observed protection against noise‐induced HL.^[^ ^340^ ^]^ In the same year, Subhendu Mukherjee and colleagues used AAV to induce brain‐derived neurotrophic factor (BDNF) expression through magnetic targeting via the RWM to prevent noise‐induced temporary HL.^[^ ^190^ ^]^

However, importantly, some studies have documented opposite results despite targeting the same gene, indicating the potential ototoxicity of AAV‐mediated gene expression without precise control. For example, one study in 2018 reported that AAV‐mediated neurotrophin‐3 (NT‐3) overexpression could mitigate noise‐induced HL.^[^ ^505^ ^]^ Conversely, Hashimoto et al. reported that forced NT‐3 overexpression can be harmful to hair cells during cochlear overstimulation.^[^ ^506^ ^]^ Similarly, AAV‐mediated delivery of glial cell line‐derived neurotrophic factor (GDNF) into the inner ear has protective effects on ototoxic drugs‐induced HL; However, contradictory findings report that GDNF overexpression can damage HCs and SGNs.^[^ ^507^, ^508^ ^]^ Furthermore, AAV‐mediated overexpression of GDNF in newborn mice causes severe neurological symptoms and HL.^[^ ^509^ ^]^ While low expression levels of AAV‐encoded proteins may be insufficient to prevent HL, excess expression can be ototoxic. The optimal balance between therapeutic expression and cytotoxicity should be precisely managed. Consequently, a thorough evaluation of the toxicity level of exogenous proteins is needed before application of these therapies. Achieving physiological levels of protein expression in sensory hair cells remains a challenge. The aim of the ongoing development of strategies such as inducible promoter manipulation is to increase such tolerability by limiting expression levels.^[^ ^510^, ^511^, ^512^ ^]^

Despite these promising developments, AAV‐mediated gene delivery for treating acquired HL remains in the early experimental stages. One challenge is that in most laboratory studies, AAV injections are administered to model animals before trauma exposure, resulting in outcomes being classified as protective effects. However, time is needed for AAV‐mediated gene transduction to take effect. In contrast, a clinical treatment scenario is more likely to occur after the trauma has occurred. The benefits of the AAV‐mediated prophylactic intervention must be weighed against the potential risks of AAV injection in populations at high risk for acquired HL.

Although various strategies have been adopted to address different inner ear diseases, these preclinical outcomes still need further evaluation. This evaluation should not only focus on safety concerns but also consider the long‐term stability of the treatments. There is no doubt that these efforts will extend the application boundaries of AAV in the inner ear.

## 6 Recent Clinical Trials of AAV for Inner Ear Diseases

Owing to the rapid development in the basic science of AAV‐mediated inner ear gene therapy, clinical trials have recently advanced. Breakthrough results continue to emerge from researchers worldwide. Recent clinical trials have adopted a gene replacement strategy in patients with OTOF mutations, one of the most common causes of nonsyndromic autosomal recessive hearing loss, also known as DFNB9.^[^ ^28^, ^29^, ^30^ ^]^ Through the dual‐AAV strategy, the large OTOF cDNA (≈6 kb) can be delivered into the inner ear through the RWM.

One recent single‐arm unilateral AAV1‐hOTOF‐mediated gene therapy in children with DFNB9 reported a significant improvement in the ABR threshold, from greater than 95 dB at baseline to 68 dB at 4 weeks (ChiCTR2200063181).^[^ ^28^ ^]^

These researchers recently amended the protocol to achieve better hearing performance, especially in noisy environments, and sound localization abilities,^[^ ^513^, ^514^ ^]^ and bilateral AAV administration was approved in their trial. They performed bilateral injections of the AAV vector through the RMW at one time to avoid the effects of neutralizing antibodies, which may reduce the efficiency of the second AAV application. Their results showed great hearing restoration in both ears in most cases patients (4/5), from >95 dB at baseline to ≈60 dB at 26 weeks, in patients aged from 1.2 to 11 years.^[^ ^29^ ^]^ Both single (5 years old) and bilateral (8 years old) cochlear AAV injections were performed by another team in China (NCT05901480).^[^ ^30^ ^]^

In another similar phase 1/2 trial sponsored by Eli Lilly's subsidiary, Akouos (NCT05572073), AK‐OTOF (Anc80‐hOTOF) was delivered through the round window membrane to an 11‐year‐old child with profound hearing loss from birth at the Children's Hospital of Philadelphia (CHOP). Thirty days after administration, hearing was restored across the tested frequencies, achieving thresholds of 65 to 20 dB HL. This study is the first conducted in the U.S. to use AAV in a clinical trial to treat hearing loss. An increasing number of clinical trials, such as NCT05788536 and NCT03996824, are being conducted.

These pioneering clinical trials represent a significant step forward. They provide invaluable knowledge about real treatment outcomes in humans. Local injection of AAV into the inner ear may pose a lower risk of systemic immune reactions than systemic AAV applications. However, despite the cautious procedures used in these trials, such as the prophylactic application of dexamethasone to minimize potential inflammatory responses, several adverse events (grade 1 or 2) have been reported.^[^ ^29^ ^]^ Additionally, early detection of the transgene cDNA in peripheral blood at one day postsurgery and a subsequent increase in the titers of neutralizing antibodies two weeks after the AAV injection have been reported.^[^ ^30^ ^]^ Long‐term, large‐scale observations are still needed to determine the long‐term effects and safety of these therapies. We also looking forward to seeing AAV applications in other inner ear gene mutation diseases beyond OTOF.

## 7 Conclusions and Future Directions

We are now at a pivotal intersection in the field of gene therapy for HL, with inspiring news emerging worldwide on research breaking through obstacles and broadening the spectrum of patients with hearing disorders that can benefit from this therapy. However, when we examine the development of AAV‐mediated gene therapy and its applications in the treatment of inner ear diseases, several challenges remain to be addressed.

First, owing to limited access to human inner ear samples, no data are available on the distribution of AAV‐infected cells throughout the human cochlea. Currently, limited data have been collected from cultured human cochlear tissue.^[^ ^143^, ^160^ ^]^ The AAV infection pattern in animals may not be the same as that in humans. Moreover, most AAV serotypes have a lower infection efficiency in OHCs than in IHCs in adult animals. This discrepancy may pose challenges in addressing external damage or inner ear mutations associated with OHCs. If the human inner ear has an AAV infection sensitivity similar to that of mice, the treatment window for inner ear applications of AAVs that target OHCs may be narrowed.

Second, in addition to CRISPR‐based correction of the genomic DNA in hair cells, which may achieve long‐term effects, the stable expression of exogenous genes carried by AAVs is directly associated with the treatment outcomes. We still do not know how long these treatment effects will last, and further observations are needed. The hearing preservation effect may fade years after the administration of AAV‐mediated gene therapy. In such cases, the readministration of AAV‐mediated gene therapy with a different serotype may need to be considered to limit the effect of NAbs.

**Figure 1:**
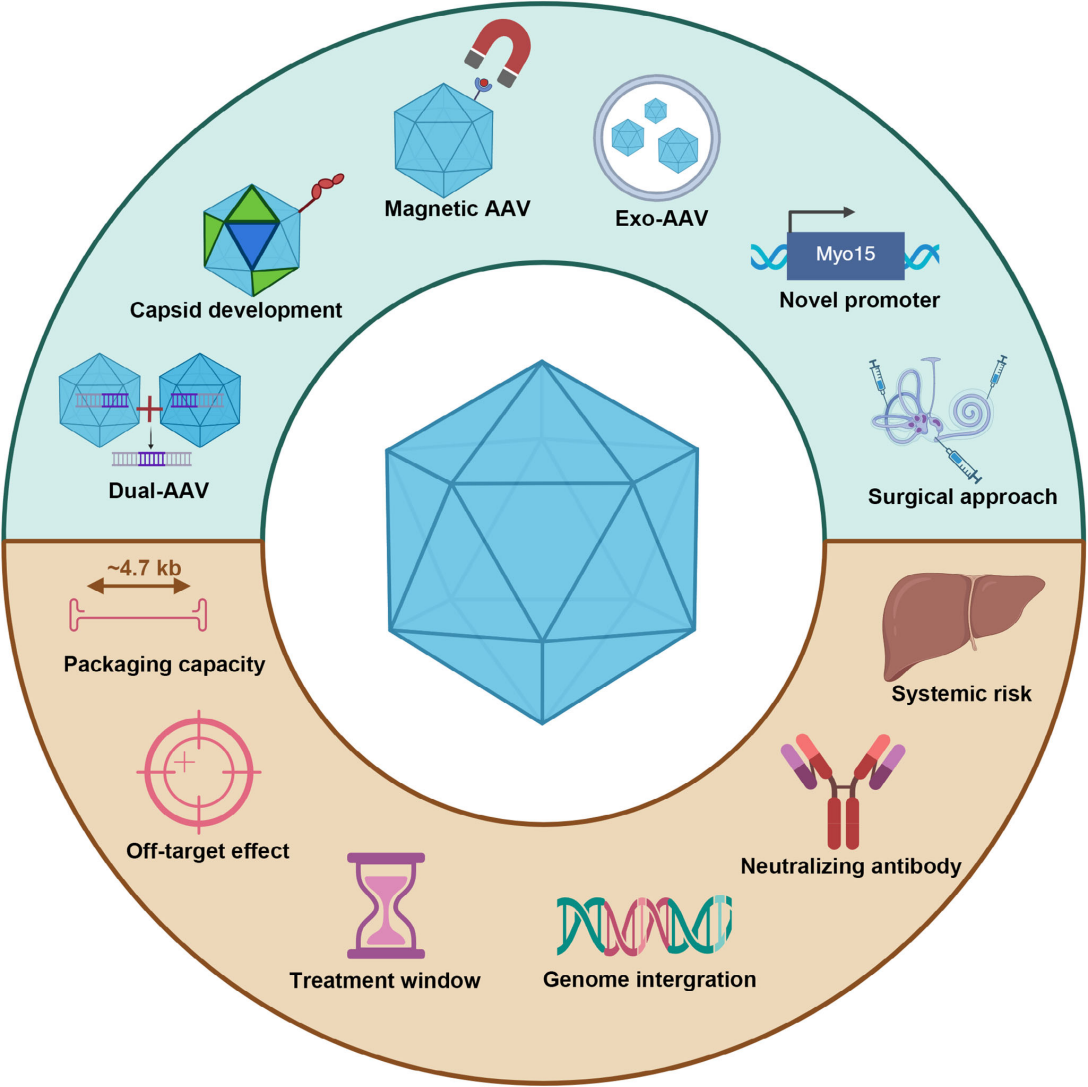
Overview of AAV development and shortcomings.

Third, off‐target effects must be considered, not only in terms of CRISPR‐associated mismatches but also in terms of unexpected AAV infections in nontarget cells. These off‐target effects could worsen treatment outcomes and increase the risks of cytotoxicity and tumorigenesis (**Figure** **1**).

Current clinical trials in AAV‐mediated gene therapy for inner ear diseases have mainly adopted a gene replacement strategy. These initial successes will encourage researchers worldwide to advance other strategies in the future. With the adoption of different strategies and the development of more precise gene‐editing tools in the future, such as more accurate gene‐editing enzymes, the range of inner ear diseases that can be treated by AAV‐mediated gene therapy will continuously expand. In addition, instead of directly changing the target sequence in the genome, the regulation (activation or inactivation) of target genes by editing the epigenome is another strategy that has been developed in recent years.^[^ ^515^ ^]^ The basic technique of this strategy is to fuse a transcriptional activator or repressor to the Cas9:sgRNA complex, which binds to the transcriptional start site of the target gene via the designated sgRNA sequence to enable increased expression of the target gene.^[^ ^516^, ^517^, ^518^, ^519^, ^520^ ^]^

Owing to the efforts of scientists worldwide, AAV‐mediated gene therapy has opened the door for patients with hereditary HL. We believe that this door will soon be open for patients with acquired HL as well. Like a double‐edged sword, AAV‐mediated gene therapy can benefit patients with HL due for various reasons while also posing risks when it is administered improperly. By proceeding cautiously, we anticipate that the development of AAV‐mediated gene‐editing technologies will bring us to the next frontier of therapeutics for the treatment of hereditary and acquired HL (**Table** **1**).

**Table 1:** Summary of studies focusing on the treatment of animal models and patients exhibiting hearing loss via inner ear gene therapy.

Type of disease	Strategy	Target gene	Model	Target cell	AAV serotype	Approach	Gene tool	Refs.
Genetic hearing loss	Gene replacement	*Vglut3*	VGLUT3 KO mice	IHC	AAV1	RWM	Stable OE	[390]
		*Vglut3*	VGLUT3 KO mice	IHC	AAV8	PSC	Stable OE	[389]
		*Otof*	*Otof* KO mice	IHC	AAV2	RWM	Stable OE	[92]
		*Otof*	*Otof* KO mice	IHC	AAV2/6	RWM	Stable OE	[261]
		*OTOF*	DFNB9 patient	IHC	N/A	RWM	Stable OE	[30]
		*OTOF*	DFNB9 patient	IHC	AAV1	RWM	Stable OE	[28]
		*Tmc1*	*Tmc*1/2 mutant mice	IHC, OHC	AAV2/1	RWM	Stable OE	[118]
		*Tmc1*	*Tmc*1/2 mutant mice	IHC, OHC	AAV1; Anc80L65	RWM	Stable OE	[401]
		*Lhfpl5*	*Lhfpl5* KO mice	IHC, OHC	exo‐AAV1/9	RWM	Stable OE	[186]
		*Kcnq1*	*Kcnq1* KO mice	SV	AAV1	RWM	Stable OE	[405]
		*Syne4*	*Syne4* KO mice	OHC	PHP.B	PSC	Stable OE	[142]
		*MsrB3*	*MsrB3* KO mice	IHC, OHC	AAV2/1	otocyst	Stable OE	[411]
		*Slc26a4*	*Slc26a4* mutant mice	cochlear epithelium	AAV2/1	otocyst	Stable OE	[410]
		*Ush1c*	*Ush1c* c.216G>A mice	IHC, OHC, VHC	AAV2/Anc80	RWM	Stable OE	[158]
		*Prestin*	*Prestin* KO mice	IHC, OHC	AAV‐ie‐K558R	RWM	Stable OE	[169]
		*Gjb2*	*Gjb2* CKO mice	cochlear epithelium	AAV1	RWM	Stable OE	[409]
		*Tmprss3*	*Tmprss3 mutant mice*	IHC, OHC, SG	AAV2	N/A	Stable OE	[408]
		*Clrn1*	*Clrn1* and *TgAC1* KO mice	IHC, OHC	AAV2, AAV8	RWM	Stable OE	[407]
		*Clrn1*	*Clrn1* KO mice	IHC, OHC	PHP.B	RWM	Stable OE	[135]
		*Clrn1*	*Clrn1* CKO mice	IHC, OHC	AAV2/8	RWM	Stable OE	[406]
	Gene disruption	*Tmc1*	Bth/+ mice (T1253A)	IHC, OHC	AAV2/9	RWM+CF	RNA interference	[422]
		*Tmc1*	Bth/+ mice (T1253A)	IHC, OHC	AAV2/Anc80	N/A	SaCas9‐KKH	[91]
		*Tmc1*	Bth/+ mice (T1253A)	IHC, OHC	PHP.eB	RWM	CasRx	[429]
		*Myo6*	*Myo6* ^WT/C442Y^ mice	IHC, OHC	PHP.eB	LW	SaCas9‐KKH	[433]
		*Kcnq4*	*Kcnq4* ^WT/G229D^ mice	OHC	PHP.eB	LW	SaCas9‐KKH	[434]
	Gene correction	*Pcdh15*	*Pcdh15* ^R245X/R245X^ mice	IHC, OHC	PHP.B	RWM	ABE8e (T‐C)	[454]
		*Pcdh15*	*Pcdh15* ^av‐3j^ mice	IHC, OHC	AAV2/9	Scala media	spCas9 (frame‐restore)	[455]
		*Myo6*	*Myo6* ^C442Y/+^ mice	IHC, OHC	PHP.eB	LW	mxABE (A‐G)	[456]
		*Otof*	*Otof* ^Q829X/Q829X^ mice	IHC	AAV‐HGHC	RWM	emxABE (A‐G)	[406]
Acquired hearing loss	Hair cell regeneration	*Atoh1*	streptomycin injuried mice	vestibular hair cell	AAV8	PSC	Stable OE	[454]
		*Net1*	neomycin injuried mice	SC	AAV‐ie	RWM	Stable OE	[173]
		Gfi1, Pou4f3, Six1, Atoh1	wild‐type mice	SC	AAV‐ie	RWM/PSC	Stable OE	[170]
		*Atoh1*	wild‐type mice	SC	AAV‐ie‐K558R	RWM	Stable OE	[169]
		*Gpm6b*	wild‐type mice; Lgr5‐GFP mice	SC	AAV‐ie	RWM	Stable OE	[172]
		*Pcolce2*	neomycin injuried mice	SC	AAV‐ie	RWM	Stable OE	[171]
		*Espin*	*Atoh1* overexpress mice	SC	AAV‐ie	RWM	Stable OE	[174]
	Prophylactic intervention	*XIAP*	Cisplatin injuried mice	OHC, SGN	AAV8‐mut733	RWM	Stable OE	[502]
		*XIAP*	Cisplatin injuried rat	OHC	AAV2	RWM	Stable OE	[501]
		*Prdx1*	Cisplatin injuried mice explant	SGN	Anc80L65	Culture medium	Stable OE	[503]
		*Htra2*	neomycin injuried mice	IHC, OHC	Anc80L65	scala media	SpCas9‐disruption	[488]
		*Htra2*	neomycin injuried mice	IHC, OHC	PHP.eB	scala media	CasRx‐disruption	[504]
		*Camkkβ*	noise‐exposed mice	OHC	AAV2.7m8	RWM	RNAi	[340]
		*Adnf9*	kanamycin injured guinea pig	IHC, OHC	AAV2	RWM	Stable OE	[507]
		*Gdnf*	kanamycin injured rat	OHC, SGN	AAV2/1	RWM	Stable OE	[508]
		*Bdnf*	noise‐exposed rat	OHC	AAV2(quad Y‐F)	RWM	Stable OE	[190]
		*Nt‐3*	noise‐exposed guinea pig	IHC, SGN	rAAV8‐mut733	scala tympani	Stable OE	[505]

IHC/OHC, inner/outer hair cell; SV, stria vascularis; SGN, spiral ganglion neuron; SC, supporting cell; RWM, round window membrane; PSC, posterior semicircular canal; CF, canal fenestration; LW, lateral wall; OE, overexpression; KO, knockout; N/A, no data.

## Conflict of Interest

The authors declare no conflict of interest.
